# Supramolecular Polymer Bottlebrushes: In Situ Assessment of Noncovalent Assemblies in Human Serum by Analytical Ultracentrifugation

**DOI:** 10.1002/marc.202400890

**Published:** 2025-02-27

**Authors:** Ilya Anufriev, Tobias Klein, Stephanie Hoeppener, Johannes C. Brendel, Ivo Nischang

**Affiliations:** ^1^ Laboratory of Organic and Macromolecular Chemistry Friedrich Schiller University Jena Humboldtstr. 10 07743 Jena Germany; ^2^ Helmholtz Institute for Polymers in Energy Applications Jena (HIPOLE Jena) Lessingstr. 12–14 07743 Jena Germany; ^3^ Jena Center for Soft Matter Friedrich Schiller University Jena Philosophenweg 7 07743 Jena Germany; ^4^ Macromolecular Chemistry I University of Bayreuth Universitätsstr. 30 95447 Bayreuth Germany; ^5^ Institute of Macromolecular Research (BIMF) and Bavarian Polymer Institute (BPI) University of Bayreuth Universitätsstr. 30 95447 Bayreuth Germany; ^6^ Helmholtz‐Zentrum Berlin für Materialien und Energie GmbH (HZB) Hahn‐Meitner‐Platz 1 14109 Berlin Germany

**Keywords:** analytical ultracentrifugation, human serum, stability, supramolecular polymer bottlebrushes

## Abstract

For nanomedical targeting and drug delivery purposes, the noncovalent assembly of polymer building blocks into defined nanostructures is an intense area of research. One of the key assets desirable to know for the potential nanocarrier is the stability under conditions close to those in application scenarios. Here, a series of polymer building blocks based on poly(ethylene glycol) (PEG), which comprise a functional end group facilitating self‐assembly into supramolecular polymer bottlebrushes (SPBs), is hydrodynamically studied. The building blocks, and consequently the assemblies, are labeled with a cyanine5 (Cy5) dye enabling selective tracing of the materials in human serum (HS) in analytical ultracentrifugation (AUC) experiments. Our experiments reveal a long‐term stability of the noncovalent assemblies over one month of storage of the materials in HS at body temperature. At the same time, the interaction of some of the Cy5 moieties with the transport protein human serum albumin (HSA) is evidenced.

## Graphical abstract

The integrity of supramolecular polymer bottlebrushes (SPBs) with envisaged stealth properties is investigated using analytical ultracentrifugation (AUC) at conditions close to the application scenario. Selective tracking of the SPBs in human serum (HS) via the Cyanine5 (Cy5) label, revealed long‐term stability at body temperature as well as interaction of dye with human serum albumin (HSA).

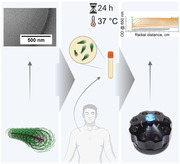

## 1 Introduction

Over the past decades, nanoscale drug delivery systems making use of suitable carrier elements such as, e.g., polymer nanoparticles (NPs) for targeted drug delivery applications are one of the modern trends in the field of experimental medicine.^[^ ^1^, ^2^ ^]^ Typically, the carrier elements are spherical in nature. However, more and more studies report the influence of the nanostructure shape on the efficacy of nanomedical targeting for drug delivery and, consequently, the potential effectiveness of medical treatment.^[^ ^3^, ^4^ ^]^ This opens the gate for interesting opportunities in the research and development of nanomaterials, such as liposomes, lipid nanodiscs, dendrimers, and exosomes.^[^ ^5^, ^6^ ^]^ Another promising nanostructure is the so‐called supramolecular polymer bottlebrushes (SPBs), which are fibrous supramolecular assemblies formed from polymer building blocks in aqueous solutions.^[^ ^7^ ^]^

One of the key parameters that such fibrous nanocarriers for biomedical applications feature is a high surface‐to‐volume ratio.^[^ ^8^, ^9^ ^]^ Elongated structures such as SPBs have a greater surface‐to‐volume‐ratio when compared to spherical NPs if the same hydrodynamic volume is assumed.^[^ ^10^ ^]^ This increases the probability of targeted interaction with potential biological interfaces exposing, e.g., cell specific ligands.^[^ ^11^, ^12^ ^]^ Moreover, studies show the potential advantages in the drug delivery enabled by such nanostructures compared to the classical spherical NPs. This concerns blood circulation time, drug loading capacity per nanocarrier, and penetration abilities for inflamed or tumor tissue.^[^ ^13^, ^14^, ^15^ ^]^

The stability of carrier elements in human serum (HS), i.e., at conditions mimicking a situation found in the human body, is an important and defining parameter of the drug delivery systems.^[^ ^16^ ^]^ Unintended interactions with proteins or simple dilution can cause disintegration of the nanocarriers and may result in undesired adverse side effects or immune activation.^[^ ^17^, ^18^ ^]^ Previously, analytical ultracentrifugation (AUC) was demonstrated to allow qualitative and quantitative investigations of the stability of nanocarrier systems under a variety of experimental conditions including varying solution composition, with the presence and absence of proteins or even in HS.^[^ ^19^ ^]^ Particularly, NPs based on biodegradable poly(lactic‐co‐glycolic acid) (PLGA) were investigated but also liposomes in HS.^[^ ^20^, ^21^ ^]^

The stability of SPBs in biological fluids has been investigated by surface plasmon resonance,^[^ ^22^ ^]^ fluorescence,^[^ ^23^ ^]^ and microscopic techniques.^[^ ^24^ ^]^ Moreover, sophisticated investigations by fluorescence correlation spectroscopy in blood samples have been reported for micellar systems.^[^ ^25^ ^]^ SPBs or related supramolecular assemblies have so far only scarcely been studied for their stability in serum or blood. Polymer nanotubes based on cyclic peptides have been tested in vivo for their blood circulation time resulting in lower half‐life times compared to covalent polymer bottlebrushes, which was explained by dynamic degradation of supramolecular assemblies.^[^ ^26^ ^]^ This example nicely illustrates how important the assessment of the stability of supramolecular systems in serum is.

Here, we investigated SPBs based on poly(ethylene glycol) (PEG) comprising a supramolecular motif as an end group, which facilitates their self‐assembly into anisotropic nanofibers. The supramolecular motifs either comprise urea or amino acid‐based units for hydrogen bond formation and hydrophobic linkers of varying lengths. On par with AUC experiments in water at 20 °C, the SPBs were studied in HS at a temperature of 37 °C to elucidate their stability and potential stealth properties.

## 2 Results and Discussion

**Scheme 1:**
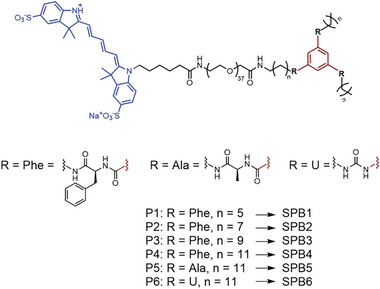
Overview of polymers P1‐P6 used as precursors for SPB1‐SPB6.

Synthetic routes of the precursors and protocols for the preparation of the SPBs have been described previously.^[^ ^27^ ^]^ The slightly modified procedures are described in the Supporting Information together with results from standard characterization techniques. The anisotropic cylindrical shapes were indicated previously via small‐angle X‐ray scattering techniques and via direct imaging with cryo‐transmission electron microscopy (cryo‐TEM) (See, Figures S1–S6, Supporting Information). The length and rigidity of the fibers are influenced by the strength of the supramolecular interactions or hydrogen bonding units, but since the aggregation follows a cooperative nucleation‐growth process, the assembly process has a significant impact on the length of the fibers, as we have reported previously.^[^ ^28^ ^]^ The chemical structure of the polymeric precursors (P1‐P6) used for self‐assembly via hydrogen bonds of the SPB1‐SPB6 can be found in **Scheme** **1**. Precursors P1–P4 have the same phenylalanine hydrogen bonding unit while having different lengths of the hydrophobic linker. Increasing the linker length increases the hydrophobic shielding of the hydrogen bonds, resulting in a potentially higher stability of the SPB. Precursors P4‐P6 have the same length as the hydrophobic linker but a varying hydrogen bonding unit comprised of phenylalanine (P4), alanine (P5), and urea (P6). The phenylalanine unit is more hydrophobic than alanine and both amino acid linkers form more hydrogen bonds per unit than urea, which can potentially lead to enhanced stability of the SPB. The functional terminus promotes self‐assembly into SPBs, while the cyanine5 (Cy5) dye moiety, with an absorbance maximum at λ=650nm, enables selective tracing of the SPBs with a minimum of optical interference from HS.^[^ ^20^, ^21^ ^]^

**Figure 1:**
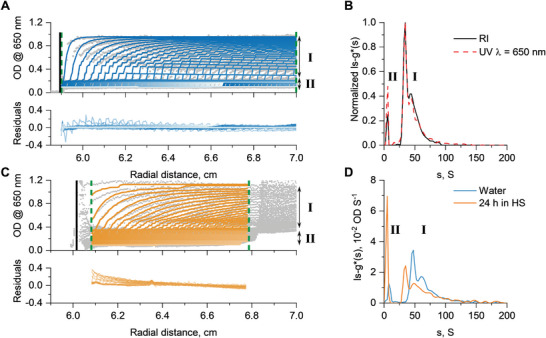
Results of the AUC investigations of SPB4 in solvents water and HS. A) Sedimentation velocity profiles utilizing the absorbance detection module in terms of optical density (OD) obtained from experiments at a rotor speed of 20 000 rpm for 24 h in water at 20 °C. Gray dots are the measured data, and colored lines are the result from the $ls-g^{*}\left(s\right)$ analysis with the residuals shown in the bottom. Profiles in region I refer to sedimenting SPBs, and region II refers to sedimenting smaller supposedly spherical micelles. B) Normalized differential distributions of sedimentation coefficients, $ls-g^{*}\left(s\right)$, of sample SPB4 in water at 20 °C obtained via RI and UV absorption optics. C) Data in analogy to Figure 1A (profiles in the region I refer to sedimenting SPBs, region II refers to sedimenting spherical micelles and to HSA interacting with Cy5 moieties). D) Comparison of differential distributions of sedimentation coefficients, $ls-g^{*}\left(s\right)$, of sample SPB4 in water and in HS after 24 h storage time at 37 °C. The black solid vertical lines in (A) and (C) indicate the positions of the meniscus, and the green dashed lines indicate the region were modeling of the data was performed.

First, a series of AUC sedimentation velocity experiments were performed in water. **Figure** **1**A shows radial distance scans during a sedimentation velocity experiment of SPB4 (the brush with the highest expected stability) in water using the absorbance optical detection system in terms of optical density (OD). In parallel, the sedimentation velocity profiles were also recorded with the universal Rayleigh Interference (RI) optics (in terms of interference fringes). Experiments were performed at an overall targeted material concentration of $c=1\;\mathrm{mg}\;ml^{-1}$ (for SPB2, the suitable concentration was found to be $c=0.5\;\mathrm{mg}\;ml^{-1}$ due to the higher dye content and otherwise leading to supersaturation of the UV detector), resulting in an appreciable OD level and signal‐to‐noise ratio in the measurements. While we chose a concentration of fibers leading to an appreciable signal‐to‐noise ratio, we also observed a concentration dependence of the apparent sedimentation coefficients in water (Figure S7, Supporting Information). The latter increased with decreased solution concentration, while the integrity of the fibers was preserved. Such an increase can be considered typical due to solution nonideality. Therefore, the comparison between HS and water solutions was always performed at the same mass concentrations with a sufficient signal‐to‐noise ratio. Numerical analysis of the experimental data in Figure 1A via the $ls-g^{*}\left(s\right)$ model was performed for the complete data range with acceptable values of residuals (i.e., small differences between experimental data and model). The distributions indicate the presence of several species as can be gauged from the sedimentation velocity data by two steps that are clearly discernable in the data (see Figure 1A,B). Interference data practically coincide with data from the absorbance optical detection module (Figure 1B). This means that data from absorbance detection are fully representative of the studied SPBs and the Cy5 is an integral component. Experiments in water show the presence of at least three resolvable colloidal species, two with s‐values in a range of 25–100 S and smaller, likely spherical micellar species sedimenting at around 5S (Figure 1A,B), all containing the Cy5 dye.^[^ ^27^, ^29^, ^30^ ^]^ The remaining plateau value of ≈0.1 OD after centrifugation at 20 000 rpm for a timescale of 24 h refers to the free Cy5 that, in separate experiments in water, did not sediment even at high rotor speeds of 42 000 rpm (Figure S8, Supporting Information). The presence of apparently free Cy5 therefore indicates some unsuccessful conjugation to the polymer precursors utilized to form the fibers. Such information is difficult to get by a mere investigation of the solutions with UV spectroscopy only and substantiates the insight made possible by AUC experiments through a combination of hydrodynamic separation and UV spectroscopic investigation.

Radial distance scans from sedimentation velocity experiments of the SPBs in HS show a more complex picture (Figure 1C) that is possible to decipher by an analogous experiment utilizing sole HS (Figure S9, Supporting Information). A significant build‐up of material at the meniscus can be observed (left from the modeled profiles in Figure 1C). This refers to the low‐density lipoprotein (LDL) fraction in HS that has been indicated in a previous study and here also (Figure S9 (Supporting Information) when compared to Figure 1C).^[^ ^20^ ^]^ Practically, the LDL fraction almost finished flotation after a few scans (at the here utilized rotor speeds). Also, there is the formation of an extended sedimentation cake (right to the modeled profiles in Figure 1C) requiring adjustment and narrowing of boundaries for modeling sedimentation velocity data. The data clearly show the persistent appearance of SPB4 and an apparent increase in the population of smaller species sedimenting around 5S. Looking at the OD values of sedimentation slices, when comparing the SPBs in water (Figure 1A) and HS (Figure 1C) suggests a slightly smaller amount of SPBs that was targeted and a slight increase in abundance of the smaller species in HS also. The analysis of data required a careful selection of radial distances for modeling (vide supra). Still, the residuals are larger when modeling typical sedimentation velocity experiments (Figure 1C bottom). Particularly, they show a drift toward smaller radial distances (left part toward the meniscus), indicating a systematic deviation of the model from the data, seen for the very first scans. This could also be due to sedimentation and flotation processes and the development of density and viscosity gradients during the AUC run (Figure 1C). However, this situation was common to all investigations with deviations in the low percentage region. Differential distributions of sedimentation coefficients, $ls-g^{*}\left(s\right)$, show very similar features from experiments performed in HS and water (Figure 1D). On average, the material sediments slower in HS than in water at 37 °C (vide infra). Integration of the differential distributions results in comparable OD values as can already be gauged from the OD of the respective SPB slice that successively sedimented to the bottom of the cell (Figure 1). These results indicate that the tested SPB is surprisingly stable in HS despite the supramolecular nature of the assemblies, which are often considered too dynamic and prone to disintegration in the presence of biological fluids.

To investigate the increased abundance of the smaller species in HS (Figure 1C,D), independent measurements of NHS‐Cy5 (used for labeling, Scheme S5, Supporting Information) in HS solution were performed. Figure S10 (Supporting Information) shows the result of NHS‐Cy5 in solution with HS at a similar molar concentration of the dye as in the respective SPB solutions. The resulting sedimentation velocity profiles and results from numerical modeling indicate the interaction of the dye with human serum albumin (HSA) as the major transport protein found in HS. Here also, differential distributions of sedimentation coefficients, c(s), utilizing the literature known partial specific volume for HSA ($\upsilon =\;\;0.73\;cm^{3}g^{-1}$)^[^ ^31^ ^]^ and modeled for both the RI and UV absorbance detection data of the Cy5 are very close regarding average sedimentation coefficients (Figure S11, Supporting Information). The results demonstrate the association of the Cy5 moiety to the HSA. Molar mass estimations for the HSA (eq. 3) from the c(s) analysis are found to be $M_{s,f}=\;66\;000\;g\;\mathrm{mo}l^{-1}$ and agree with the literature ($s=4.56\;S,\;f/f_{sph}=1.28$).^[^ ^31^ ^]^ Therefore, NHS‐Cy5 binds to the protein.^[^ ^32^ ^]^

As exemplified for SPB4, the remaining SPBs (Scheme 1) are investigated analogously. Figure S12 (Supporting Information) shows results from sedimentation velocity experiments of all SPBs in purely aqueous solutions at a temperature of 20 °C. The differential distributions of sedimentation coefficients, $ls-g^{*}\left(s\right)$, show a wide range of sedimentation coefficients, indicating rather significant dispersity. In some cases, the heterogeneity of the populations of colloidal material is observed. The presence of assemblies with different lengths is also indicated by cryo‐TEM images (see SI). In instances, a minor fraction of colloidal species of smaller sedimentation coefficients, also containing Cy5, is observed (Figure S12, Supporting Information). The occurrence of a broad dispersity in the samples and different fiber sizes is only partially related to the strength of the hydrogen bonds formed, but severely impacted by the assembly process as described previously.^[^ ^28^ ^]^ The resulting fibers appear kinetically trapped and nucleation and growth decide mostly on the final fiber distribution. Therefore, several populations can be considered typical for the respective materials, particularly when investigated by sedimentation velocity AUC experiments.^[^ ^27^, ^29^, ^30^ ^]^ Dynamic light scattering (DLS) tends to discriminate against smaller colloidal species also.

To exemplify colloidal stability of the fibers undergoing AUC experiments, cells were shaken for 10 min after a completed sedimentation velocity run at 20 000 rpm for 24 h. Afterward, the cells were aligned in the rotor and another sedimentation velocity experiment was performed including the same analytical procedure. This led to practically coinciding differential distributions of sedimentation coefficients, $ls-g^{*}\left(s\right)$, obtained from modeling experimental sedimentation velocity data from both experiments. This indicates that the SPBs are not altered in the centrifugal field and by compacting them to the cell bottom in AUC experiments (Figure S13, Supporting Information). Also, throughout this study we noticed that, over a timescale of several months, independent experiments led to similar differential distributions of sedimentation coefficients, $ls-g^{*}\left(s\right)$, indicating that the supramolecular structure is not affected by storage times over several months as exemplified for SPB4 in water (Figure S14, Supporting Information).

**Figure 2:**
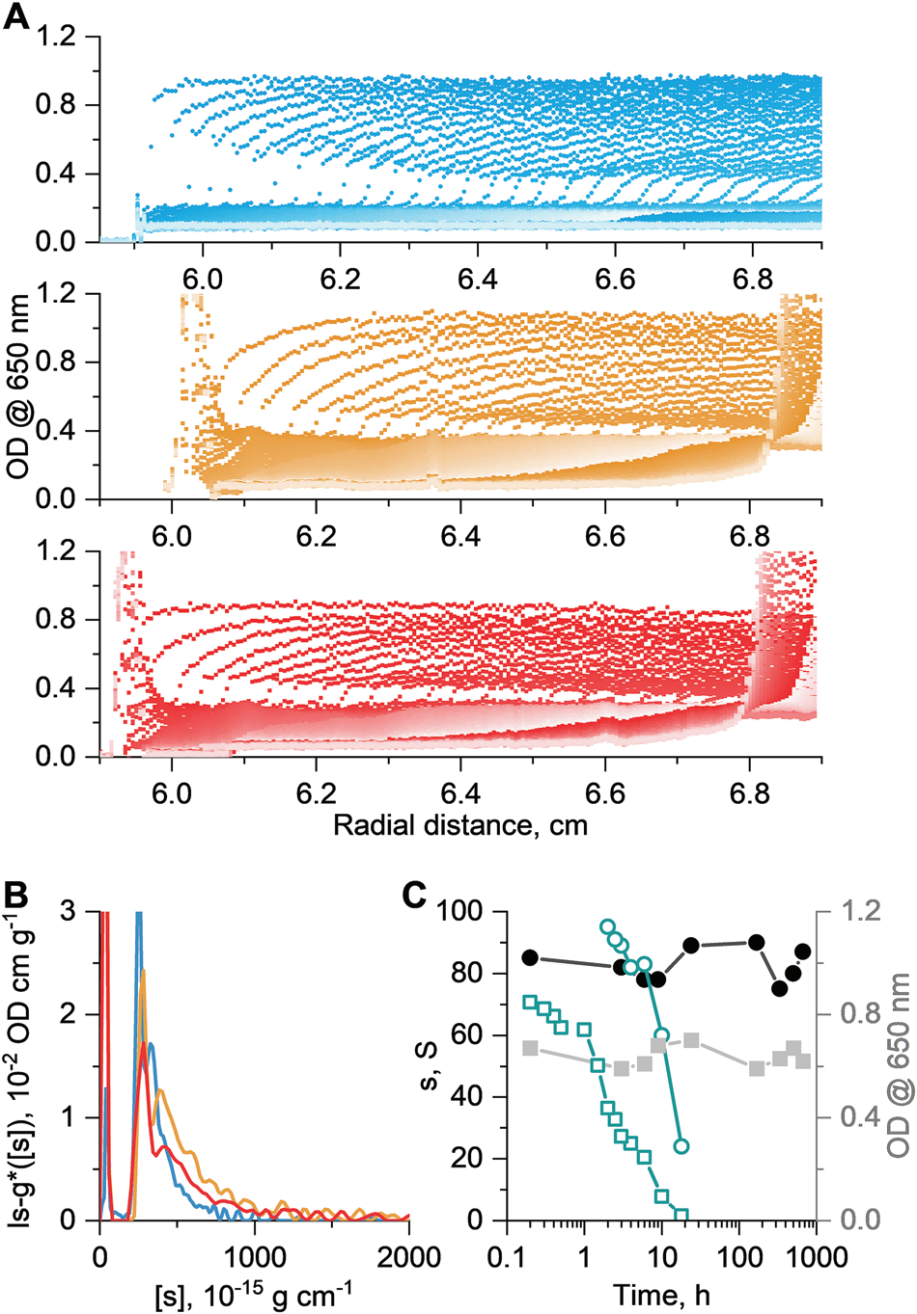
Results of AUC investigations of SPB4 in HS. A) Evolution of sedimentation velocity profiles with time during the sedimentation velocity experiment in water (blue color) and after storage in HS for 24 h (yellow color) and 1‐month (red color), B) differential distributions of intrinsic sedimentation coefficients, $ls-g^{*}\left(\left[s\right]\right)$, from modeling of sedimentation velocity profile scans shown in Figure 2A, and C) signal (weight) average value of sedimentation coefficients (conneted black circles), and OD (connected gray squares) for SPBs stored in HS for different times. The connected cyan symbols refer to the literature in which PLGA NPs have been investigated in HS with the OD stemming from the dye moiety contained in the PLGA NPs at λ=635nm (connected cyan squares) and the signal (weight) average value of sedimentation coefficients of the NPs (connected cyan circles) from storage experiments in HS.^[^ ^20^ ^]^

After characterizing the sedimentation behavior in water, we performed experiments in HS in analogy to the results presented in Figure 1. At first, we performed stability tests for SPB4 with a storage time of 9 h in HS at 37 °C. Except for a noticeable shift toward overall smaller sedimentation coefficients, no notable changes in the overall OD level and differential distributions of sedimentation coefficients, $ls-g^{*}\left(s\right)$, were observed upon varying storage times in HS (Figure S15, Supporting Information). This shift toward smaller sedimentation coefficients could be explained by the increased viscosity, $\eta_{0}$, and density, $\rho_{0}$ of HS solutions when compared to water only (see Table S1, Supporting Information). Thus, we decided to elongate the storage time of all samples to 24 h. Still, there is no significant difference in both the features of the differential distribution of sedimentation coefficients, $ls-g^{*}\left(s\right)$, and the overall OD values visible, indicating stability under readily long exposure times (Figure S16, Supporting Information). For sample SPB4, we also performed stability studies with a storage time of up to one month with samples investigated within 1‐week time intervals (**Figure** **2**A). To facilitate better comparisons between results obtained in water and HS, we converted the differential distributions of sedimentation coefficients, $ls-g^{*}\left(s\right)$, to differential distributions of intrinsic sedimentation coefficients, $ls-g^{*}\left(\left[s\right]\right)$ (eq. 1). [s] considers the solvent's viscosity, $\eta_{0}$, and density, $\rho_{0}$, allowing us to compare sedimentation inevitably performed in differently dense and viscous solvents. Even at such an extended period of storage time in HS, we could observe an intrinsic sedimentation behavior (eq. 1) from the SPBs appearing overall similar in water and serum, however with a trend of some broadening toward larger [s]‐values in serum (Figure 2B). Throughout the entire time, the sedimentation coefficient and OD level of the SPB4 in HS on average take values of s=83±5S and OD =0.64±0.04, respectively. No systematic trend in the data was observed.

In the quest to understand the unusually high stability of the fibers, we compare the SPBs in HS with biodegradable PLGA NPs from a previous study (Figure 2C).^[^ ^20^ ^]^ The overall abundance of the NPs (in terms of overall OD) as traced by the cell‐targeting dye moiety, decreased rapidly once in contact with HS at 37 °C, already below a time scale of 1 h.^[^ ^20^ ^]^ For example, an OD value of 0.8 decreased to below 0.2 within 10 h. The rapid decrease in OD was accompanied by a rapid decrease in the sedimentation coefficients from more than 90 to less than 30 only. This indicates successive erosion of the PLGA nanocarrier over time with a fade of material while storing in HS. In contrast, the SPBs still appear unaffected after hundreds of hours. The data demonstrates, under a comparable set of conditions, the stability of the SPBs assembled from non‐degradable polymer building blocks. This stability is believed to be essential for the envisaged stealth properties.

Having demonstrated the apparently high stability of the SPB4, when compared to medical NPs made from the gold‐standard pharmapolymer PLGA, we take a closer look at the complex set of data.

**Figure 3:**
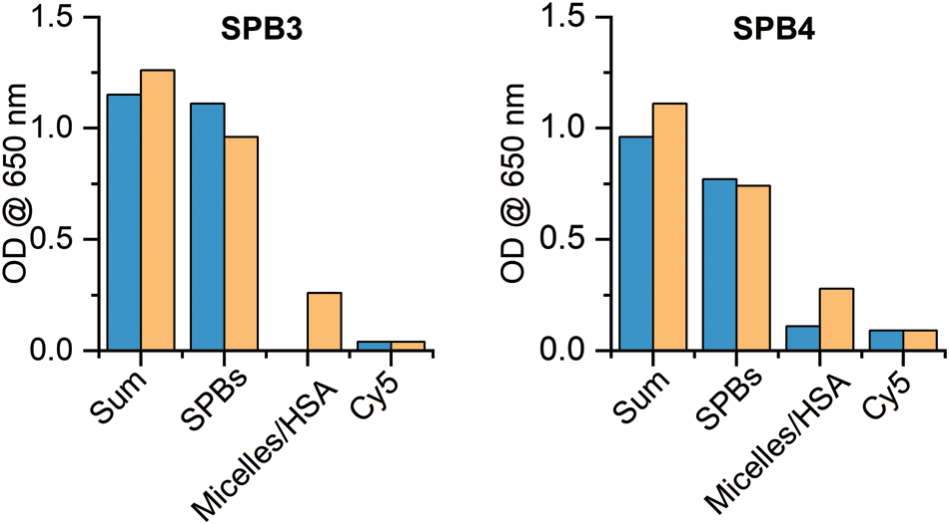
Bar charts from the OD level at λ=650 nm of the different components originating from the SPB3 and SPB4 samples in water (blue bars) and after 24 h of storage at T  =  37 °C in HS (orange bars) before the respective AUC experiments.

**Figure** **3** shows bar charts of the OD level of the components of all SPBs in the solution. We here focus on the signal OD assignable to the different components that can be identified from sedimentation velocity runs of the SPBs in water (blue bars) and in HS (orange bars). The data were acquired after 24 h of storage at 37 °C in HS.

Keeping in mind that each sample was prepared separately, the experimental error by individually preparing solution concentrations in HS, could result in slight variations in OD levels (vide supra). The overall signal from the sample in HS solutions is slightly larger compared to water and on average increases by 15%(±7) when averaging from all SPBs. This is due to the increase in the refractive index of the HS solution. An increase in refractive index due to the presence of HS leads to additional scattering of light. The OD originating from fibers has a lower level in HS compared to water. The Cy5 signal monitored in the range of the spherical micelles’ sedimentation coefficients significantly increases in HS solutions. If the micelles were initially absent, sedimentation coefficients in this range were observed. This indicates the presence of Cy5 moieties in newly formed species. Those can only originate from SPBs when exposing them to HS. In other words, HSA extracts Cy5 moieties from the fibers (likely also the pendant polymers) without affecting the integrity of the SPBs. The latter indicates that dynamic processes may still occur despite the overall stability of the fibers. The OD level of free Cy5 does not change significantly in all cases.

Results for the other SPBs are shown in the Figure S17 (Supporting Information). Quantitatively, there are differences among the SPBs, however, representing the overall same qualitative behavior in HS.

## 3 Conclusion

We have demonstrated the investigation of anisotropic SPBs and their behavior in HS when compared to their simple aqueous solutions only. For this purpose, the materials were investigated in situ in solution with HS. The experiments are possible without the need for complex processing steps, are globally dilution free, and operate under conditions of a conserved mass balance in the hermetically closed system. Our experiments demonstrate the unusually high stability of the SPBs for rather long periods of time in HS as a requirement for stealth properties. This is regardless of the bonding strength of assembly‐promoting moieties within the systems. The significance of the observations made, was underpinned by recent studies that identified immediate changes to gold‐standard PLGA NPs after contact with HS. Conclusively, there is a significant increase in the overall optical density when diluting SPBs in HS as compared to water. Commonly, the interaction of Cy5 moieties with HSA was evidenced in all systems. This approach to the hydrodynamic investigation of nanostructures provides relevant information about the systems, which should be considered before direct in vivo experiments for determining the stability of materials. With the provided data at hand, the fingerprint of complex nanoscale drug delivery systems in HS can be utilized to obtain advanced information useful for their application as drug carrier elements.

## 4 Experimental Section

AUC sedimentation velocity experiments were performed with an Optima Analytical Ultracentrifuge (Beckman Coulter, Brea, CA). The cells were assembled with double‐sector Epon centerpieces with a 12 mm optical solution path length and were filled with ca. 440 µL of pure solvent, i.e., water, in the reference sector and ca. 420 µL of SPB1‐6 solutions (assembled from P1‐P6) in the sample sector. Assembled cells were placed in an eight‐hole rotor (An‐50Ti). Sedimentation velocity experiments were conducted in a multispeed setting. As a first step, a rotor speed of 20 000 rpm for 24 h was utilized, followed by a speed ramp toward 42 000 rpm and spinning at that speed for another 24 h. Sedimentation velocity profile scans were recorded with the interference optical detection system (refractive index (RI)) and with absorbance detection in terms of optical density (OD) at the wavelength of the Cy5 dye (λ=650 nm). Experiments in water and D_2_O were conducted at a temperature of 20 °C and in water and HS at a temperature of 37 °C (the determined solvent properties in terms of density, $\rho_{0}$, and viscosity, $\eta_{0}$, were listed in Table S1, Supporting Information). To mimic conditions in the human body, HS was diluted with water to an overall of 82.6 wt.%. Fiber sample solutions in HS were prepared such that the total serum content of 55 wt.% at a fiber sample concentration of $c=1\;\mathrm{mg}\;ml^{-1}$ was obtained.^[^ ^33^ ^]^ Each fiber solution was prepared separately, i.e., not in a large batch, which has to be considered when comparing experimental results. For stability studies, AUC cells were filled with the samples in the sample sector and water in the reference sector (vide supra). They were placed in the rotor and incubated in the oven (Memmert, Schwabach, Germany) at a temperature of 37 °C for the desired time. Afterward, they were put in the preheated AUC chamber, thereby ensuring a minimum time window outside the desired incubation temperature. Care was taken that this process was the same for all samples. Data evaluation was performed by using the software SEDFIT (version 16.1) via modeling with the least squares boundary, i.e., $ls-g^{*}\left(s\right)$ analysis without considering the effects of diffusion.^[^ ^34^ ^]^ In instances, sedimentation‐diffusion analysis by numerical solution of the Lamm equation via the c(s) model was used.^[^ ^35^ ^]^ The partial specific volumes, υ, from experiments were determined by density variation sedimentation velocity experiments in water and D_2_O/water mixtures whose density and viscosity have also been determined (Table S1, Supporting Information).^[^ ^36^ ^]^ For the determination, the introduction of the intrinsic sedimentation coefficient appears useful:

(1) $$\left[s\right]=\frac{s\eta_{0}}{1-\upsilon \rho_{0}}\;$$

where $\eta_{0}$ and $\rho_{0}$ are the density and viscosity of the solvent. The partial specific volumes, υ, from experiments can then be determined by the measured viscosity and density values of water and the D_2_O/water mixtures and by equating [s] for the two solvents. This leads to the partial specific volume, υ, calculated after the following equation:^[^ ^36^ ^]^

(2) $$\upsilon =\frac{s_{2\;}\eta_{2}-s_{1\;}\eta_{1}}{s_{2\;}\eta_{2}\rho_{1}-s_{1\;}\eta_{1}\rho_{2}}$$

where $s_{1\;}$, $\eta_{1}$, and *ρ*_1_ are the sedimentation coefficient, viscosity, and density of one solvent composition of the pair (e.g., water) and $s_{2\;}$, $\eta_{2}$, and *ρ*_2_ from the respective other composition (e.g., a water / D_2_O mixture). The determined υ‐values are listed in Table S2 (Supporting Information). Knowledge of [s] also allows for the calculation of molar masses based on sedimentation‐diffusion analysis:^[^ ^19^ ^]^

(3) $$M_{s,f}=9\pi \sqrt{2}N_{A}{\left(\left[s\right]\left(f/f_{\mathrm{sph}}\right)\right)}^{3/2}\sqrt{\upsilon }\;$$

where $N_{A}$ is the Avogadro number and $\;f/f_{sph}$ is the translational frictional ratio.

## Conflict of Interest

The authors declare no conflict of interest.

## Supporting information



Supporting Information

## Data Availability

The data that support the findings of this study are available in the supplementary material of this article.
