# Supplementary material for: Supramolecular Polymer Bottlebrushes: In Situ Assessment of Noncovalent Assemblies in Human Serum by Analytical Ultracentrifugation
Source: Macromol Rapid Commun. 2025 Feb 27;46(10):2400890. doi: 10.1002/marc.202400890 (PMC12087737; doi:10.1002/marc.202400890)
Supplement: Supplementary file 1 — Supporting Information [file MARC-46-2400890-s001.docx]

Supporting Information

Supramolecular Polymer Bottlebrushes: In Situ Assessment of Noncovalent Assemblies in Human Serum by Analytical Ultracentrifugation

Ilya Anufriev, Tobias Klein, Stephanie Hoeppener, Johannes C. Brendel, and Ivo Nischang*

[1. Synthesis S2](#_Toc148966758)

[1.1 Synthetic routes to core moieties (Schemes S1-S6) S2](#_Toc148966759)

[1.2 Synthetic routes to polymer fiber precursors S4](#_Toc148966760)

[2. Characterization S](#_Toc148966761)[11](#_Toc148966762)

[2.2 Analytical ultracentrifugation (AUC) (Figures S7-S17 & Tables S1, S2, Eq. S1) S17](#_Toc148966763)

[3. References S2](#_Toc148966764)3

# Synthesis

## Synthetic routes to core moieties

The synthetic routes to the core moieties were performed according to previously published procedures without any changes.^[1, 2]^





Scheme S1. Synthetic route to the benzenetrispeptide conjugates 13-17 bearing a Boc-protected PEO chain.





Scheme S2. Synthetic route to the benzenetrisurea conjugate 18 bearing a Boc-protected PEO chain.





Scheme S3. Boc-deprotection of 13-17 resulting in 19-23.





Scheme S4. Boc-deprotection of 18 resulting in 24.





Scheme S5. Attachment of NHS-Cy5 to 19-23 resulting in 1-5.





Scheme S6. Attachment of NHS-AF488 to 24 resulting in 6.

## Synthetic routes to polymer fiber precursors

**Conjugation of NHS-PEO_2k_-NHBoc**

P1: [B][Phe]_3_[C_6_]_3_[PEO_2k_-NHBoc] **13**

The synthesis was adapted from previously published procedures.^[1, 2]^ The starting material **7** (0.1 g, 0.097 mmol, 1.0 eq) was dissolved in DMF. To this solution, 135 µL of triethylamine (0.97 mmol, 10 eq) were added and the solution stirred for 15 min at room temperature (rt). Subsequently, NHS-PEO_2k_-NHBoc (0.25 g, 1.35 eq) was added and the mixture stirred for 24 h at rt. After checking the conversion via SEC, the reaction mixture was precipitated into cold diethyl ether and subsequently centrifuged. The supernatant was discarded and an Et2O:acetone (9:1 v:v) solution added to the precipitate to remove residual PEO traces. After another centrifugation, the supernatant was decanted and the precipitate dried in vacuo (40 °C) overnight. The obtained white solid was dissolved in MilliQ water (3 mg/mL) by stirring vigorously overnight. Residual PEO traces could be removed via several centrifugal washing steps using Amicon centrifugal filter units (10 kDa MWCO). The retained solution was then freeze dried overnight to yield a white powder.

**Yield:** 262 mg, 0.08 mmol (82%), white powder.

**^1^H-NMR (300 MHz, d_6_-DMSO, 298 K):** *δ* [ppm] = 8.73 (t, J = 7.2 Hz, 3H, NH), 8.3 (m, 3H, CH_aromat_), 8.10 (s, 3H, NH), 7.38 – 7.10 (m, 15H, CH_aromat_), 6.81 – 6.72 (m, 1H, NH), 4.78 – 4.66 (m, 3H, CH), 4.07 – 4.00 (m, 2H, CH_2_), 3.77 – 3.71 (m, 2H, CH_2_), 3.50 (m, 148H, PEO), 3.15 – 2.88 (m, 14H, CH_2_), 1.37 (m, 14H, CH_2_), 1.22 (m, 16H, CH_2_), 0.84 (t, J = 6.4 Hz, 6H, CH_3_).

**SEC (DMAc + 0.21 wt.% LiCl):** *M*_n_ = 4,200 g mol^-1^_;_ *M*_w_ = 4,600 g mol^-1^_;_ *Đ* = 1.09.

P2: [B][Phe]_3_[C_8_]_3_[PEO_2k_-NHBoc] **14**

**14** was synthesized according to the procedure of compound **13**.

**Yield:** 126 mg, 0.045 mmol (84%), white powder.

**^1^H-NMR (300 MHz, d_6_-DMSO, 298 K):** δ [ppm] = 8.71 (t, J = 7.2 Hz, 3H, NH), 8.29 (m, 3H, CH_aromat_), 8.09 (s, 3H, NH), 7.38 – 7.10 (m, 15H, CH_aromat_), 6.75 (s, 1H, NH), 4.79 – 4.64 (m, 3H, CH), 4.07 – 3.98 (m, 2H, CH_2_), 3.73 (m, 148H, PEO), 3.14 – 2.86 (m, 14H, CH_2_), 1.37 (m, 14H, CH_2_), 1.22 (m, 28H), 0.83 (t, J = 6.4 Hz, 6H, CH_3_).

**SEC (DMAc + 0.21 wt.% LiCl):** M_n_ = 3,600 g mol^-1^_;_ M_w_ = 3,900 g mol^-1^_;_ Đ = 1.07.

P3: [B][Phe]_3_[C_10_]_3_[PEO_2k_-NHBoc] **15**

**15** was synthesized according to the procedure of compound **13**.

**Yield:** 118 mg, 0.041 mmol (76%), white powder.

**^1^H-NMR (300 MHz, d_6_-DMSO, 298 K):** δ [ppm] = 8.71 (t, J = 6.6 Hz, 3H, NH), 8.29 (m, 3H, CH_aromat_), 8.09 (s, 3H, NH), 7.37 – 7.08 (m, 15H, CH_aromat_), 6.75 (s, 1H, NH), 4.77 – 4.66 (m, 3H, CH), 4.02 (s, 2H, CH_2_), 3.73 (m, 2H, CH_2_), 3.50 (m, 148H, PEO), 3.14 – 2.87 (m, 14H, CH_2_), 1.37 (m, 14H, CH_2_), 1.21 (m, 40H), 0.83 (t, J = 6.4 Hz, 6H, CH_3_).

**SEC (DMAc + 0.21 wt.% LiCl):** M_n_ = 3,900 g mol^-1^_;_ M_w_ = 4,100 g mol^-1^_;_ Đ = 1.06.

P4: [B][Phe]_3_[C_12_]_3_[PEO_2k_-NHBoc] **16**

**16** was synthesized according to the procedure of compound **13**.

**Yield:** 118 mg, 0.040 mmol (79%), white powder.

**^1^H-NMR (300 MHz, d_6_-DMSO, 298 K):** δ [ppm] = 8.71 (t, J = 7.3 Hz, 3H, NH), 8.35 – 8.23 (m, 3H, CH_aromat_), 8.09 (s, 3H, NH), 7.35 – 7.10 (m, 15H, CH_aromat_), 6.75 (t, J = 5.4 Hz, 1H, NH), 4.76 – 4.65 (m, 3H, CH), 4.06 – 3.99 (m, 2H, CH_2_), 3.77 – 3.69 (m, 2H, CH_2_), 3.50 (m, 148H, PEO), 3.13 – 2.87 (m, 14H, CH_2_), 1.37 (m, 14H, CH_2_), 1.21 (m, 52H), 0.88 – 0.78 (m, 6H, CH_3_).

**SEC (DMAc + 0.21 wt.% LiCl):** M_n_ = 4,000 g mol^-1^_;_ M_w_ = 4,200 g mol^-1^_;_ Đ = 1.05.

P5: [B][Ala]_3_[C_12_]_3_[PEO_2k_-NHBoc] **17**

**17** was synthesized according to the procedure of compound **13**.

**Yield:** 197 mg, 0.072 mmol (76%), white powder.

**^1^H-NMR (300 MHz, d_6_-DMSO, 298 K):** δ [ppm] = 8.66 (d, J = 7.3 Hz, 3H, NH), 8.47 (s, 3H, CH_aromat_), 7.95 (t, J = 5.2 Hz, 3H, NH), 7.17 (t, J = 5.6 Hz, 1H, NH), 6.83 – 6.70 (m, 1H, NH), 4.56 – 4.41 (m, 3H, CH_2_), 4.09 – 3.97 (m, 2H, CH_2_), 3.80 – 3.70 (m, 1H), 3.50 (m, 148H, PEO), 3.05 (q, J = 5.5 Hz, 8H, CH_2_), 2.98 – 2.87 (m, 2H, CH_2_), 1.36 (m, 14H, CH_2_), 1.22 (m, 52H), 0.84 (t, J = 6.6 Hz, 6H, CH_3_).

**SEC (DMAc + 0.21 wt.% LiCl):** M_n_ = 3,200 g mol^-1^_;_ M_w_ = 3,500 g mol^-1^_;_ Đ = 1.09.

P6: [B][U]_3_[C_12_]_3_[PEO_2k_-NHBoc] **18**

**18** was synthesized according to the procedure of compound **13**.

**Yield:** 197 mg, 0.072 mmol (76%), white powder.

**^1^H-NMR (300 MHz, d_6_-DMSO, 298 K):** δ [ppm] = 8.66 (d, J = 7.3 Hz, 3H, NH), 8.47 (s, 3H, CH_aromat_), 7.95 (t, J = 5.2 Hz, 3H, NH), 7.17 (t, J = 5.6 Hz, 1H, NH), 6.83 – 6.70 (m, 1H, NH), 4.56 – 4.41 (m, 3H, CH_2_), 4.09 – 3.97 (m, 2H, CH_2_), 3.80 – 3.70 (m, 1H), 3.50 (m, 148H, PEO), 3.05 (q, J = 5.5 Hz, 8H, CH_2_), 2.98 – 2.87 (m, 2H, CH_2_), 1.36 (m, 14H, CH_2_), 1.22 (m, 52H), 0.84 (t, J = 6.6 Hz, 6H, CH_3_).

**SEC (DMAc + 0.21 wt.% LiCl):** M_n_ = 3,000 g mol^-1^_;_ M_w_ = 3,400 g mol^-1^_;_ Đ = 1.13.

P1: [B][Phe]_3_[C_6_]_3_[PEO_2k_-NH_3_] **19**

Compound **13** (0.22 g, 0.081 mmol, 1.0 eq) was dissolved in 1.628 mL DCM. To this, 262 µL of an 18:1:1 solution of TFA/TIPS/H_2_O (TFA: 226 µL, 2.93 mmol, 36 eq; TIPS: 33 µL, 0.163 mmol, 2 eq; H_2_O: 2.9 µL, 0.163 mmol, 2 eq) was added dropwise and the solution stirred for 2 h at rt. Afterwards, half of the DCM volume was removed and the concentrated reaction mixture precipitated in cold diethyl ether. The suspension was centrifuged (3 min, 8,000 rpm) and the supernatant decanted. The obtained product was dried in the vacuum oven (40 °C overnight).

**Yield:** 221 mg, 0.077 mmol (95%), white solid.

**^1^H-NMR (300 MHz, d_6_-DMSO, 298 K):** δ [ppm] = 8.72 (t, J = 7.0 Hz, 3H, NH), 8.37 – 8.24 (m, 3H, CH_aromat_), 8.18 – 8.04 (m, 3H, NH), 7.71 (s, 2H, NH_2_), 7.37 – 7.09 (m, 15H, CH_aromat_), 4.79 – 4.64 (m, 3H, CH), 4.08 – 3.97 (m, 2H, CH_2_), 3.78 – 3.70 (m, 2H, CH_2_), 3.50 (m, 148H, PEO), 3.17 – 2.86 (m, 14H, CH_2_), 1.44 – 1.30 (m, 8H, CH_2_), 1.30 – 1.13 (m, 16H, CH_2_), 0.84 (t, J = 6.5 Hz, 6H, CH_3_).

**SEC (DMAc + 0.21 wt.% LiCl):** M_n_ = 3,400 g mol^-1^_;_ M_w_ = 4,100 g mol^-1^_;_ Đ = 1.19.

P2: [B][Phe]_3_[C_8_]_3_[PEO_2k_-NH_3_] **20**

**20** was synthesized according to the procedure of compound **19**.

**Yield:** 179 mg, 0.064 mmol (91%), white solid.

**^1^H-NMR (300 MHz, d_6_-DMSO, 298 K):** δ [ppm] = 8.79 – 8.68 (m, 3H, NH), 8.37 – 8.24 (m, 3H, CH_aromat_), 8.20 – 8.04 (m, 3H, NH), 7.70 (s, 2H, NH_2_), 7.39 – 7.07 (m, 15H, CH_aromat_), 4.83 – 4.62 (m, 3H, CH), 4.08 – 3.96 (m, 2H, CH_2_), 3.80 – 3.70 (m, 2H, CH_2_), 3.50 (m, 148H, PEO), 3.18 – 2.86 (m, 14H, CH_2_), 1.43 – 1.29 (m, 8H, CH_2_), 1.29 – 1.13 (m, 28H, CH_2_), 0.91 – 0.76 (m, 3H, CH_3_).

**SEC (DMAc + 0.21 wt.% LiCl):** M_n_ = 3,700 g mol^-1^_;_ M_w_ = 4,100 g mol^-1^_;_ Đ = 1.11.

P3: [B][Phe]_3_[C_10_]_3_[PEO_2k_-NH_3_] **21**

**21** was synthesized according to the procedure of compound **19**.

**Yield:** 204 mg, 0.071 mmol (93%), white solid.

**^1^H-NMR (300 MHz, d_6_-DMSO, 298 K):** δ [ppm] = 8.78 – 8.66 (m, 3H, NH), 8.37 – 8.22 (m, 3H, CH_aromat_), 8.09 (t, J = 6.6 Hz, 3H, NH), 7.69 (s, 2H, NH_2_), 7.38 – 7.05 (m, 15H, CH_aromat_), 4.70 (dt, J = 8.9, 5.3 Hz, 3H, CH), 4.07 – 3.96 (m, 2H, CH_2_), 3.78 – 3.68 (m, 2H, CH_2_), 3.61 – 3.45 (m, 148H. PEO), 3.12 – 2.86 (m, 14H, CH_2_), 1.45 – 1.29 (m, 8H, CH_2_), 1.29 – 1.10 (m, 40H, CH_2_), 0.83 (t, J = 6.2 Hz, 6H, CH_3_).

**SEC (DMAc + 0.21 wt.% LiCl):** M_n_ = 3,900 g mol^-1^_;_ M_w_ = 4,300 g mol^-1^_;_ Đ = 1.11.

P4: [B][Phe]_3_[C_12_]_3_[PEO_2k_-NH_3_] **22**

**22** was synthesized according to the procedure of compound **19**.

**Yield:** 181 mg, 0.061 mmol (86%), white solid.

**^1^H-NMR (300 MHz, d_6_-DMSO, 298 K):** δ [ppm] = 8.71 (t, J = 6.7 Hz, 3H, NH), 8.36 – 8.22 (m, 3H, CH_aromat_), 8.17 – 8.03 (m, 3H, NH), 7.70 (s, 2H, NH_2_), 7.36 – 7.06 (m, 15H, CH_aromat_), 4.80 – 4.60 (m, 3H, CH), 4.02 (t, J = 4.8 Hz, 2H, CH_2_), 3.72 (d, J = 5.2 Hz, 2H, CH_2_), 3.63 – 3.45 (m, 148H, PEO), 3.13 – 2.85 (m, 14H), 1.43 – 1.29 (m, 8H, CH_2_), 1.29 – 1.09 (m, 52H, CH_2_), 0.83 (t, J = 6.2 Hz, 6H, CH_3_).

**SEC (DMAc + 0.21 wt.% LiCl):** M_n_ = 3,900 g mol^-1^_;_ M_w_ = 4,600 g mol^-1^_;_ Đ = 1.17.

P5: [B][Ala]_3_[C_12_]_3_[PEO_2k_-NH_3_] **23**

**23** was synthesized according to the procedure of compound **19**.

**Yield:** 173 mg, 0.063 mmol (87%), white solid.

**^1^H-NMR (300 MHz, d_6_-DMSO, 298 K):** δ [ppm] = 8.65 (d, J = 5.1 Hz, 3H, NH), 8.47 (d, J = 4.5 Hz, 3H, CH_aromat_), 7.96 (d, J = 4.9 Hz, 3H, NH), 7.69 (s, 2H, NH_2_), 7.26 – 7.11 (m, 1H, NH), 4.58 – 4.38 (m, 3H, CH), 4.10 – 3.93 (m, 2H, CH_2_), 3.74 (d, J = 3.6 Hz, 2H, CH_2_), 3.67 – 3.44 (m, 148H, PEO), 3.13 – 2.83 (m, 8H, CH_2_), 1.47 – 1.29 (m, 14H, CH_2_), 1.29 – 1.11 (m, 52H, CH_2_), 0.84 (t, J = 5.7 Hz, 6H, CH_3_).

**SEC (DMAc + 0.21 wt.% LiCl):** M_n_ = 3,500 g mol^-1^_;_ M_w_ = 3,800 g mol^-1^_;_ Đ = 1.08.

P6: [B][U]_3_[C_12_]_3_[PEO_2k_-NH_3_] **24**

**24** was synthesized according to the procedure of compound **19**.

**Yield:** 76 mg, 0.003 mmol (99%), white solid.

**^1^H-NMR (300 MHz, d_6_-DMSO, 298 K):** δ [ppm] = 8.26 (s, 3H, CH_aromat_), 7.69 (s, 2H, NH_2_), 7.17 (t, J = 5.2 Hz, 1H, NH), 7.08 (s, 3H, NH), 5.94 (t, J = 4.9 Hz, 3H, NH), 4.07 – 3.96 (m, 2H, CH_2_), 3.73 (d, J = 4.9 Hz, 2H, CH_2_), 3.65 – 3.43 (m, 148H, PEO), 3.10 – 2.86 (m, 8H, CH_2_), 1.50 – 1.32 (m, 8H, CH_2_), 1.32 – 1.13 (m, 52H, CH_2_), 0.85 (t, J = 6.6 Hz, 6H, CH_3_).

**SEC (DMAc + 0.21 wt.% LiCl):** M_n_ = 3,800 g mol^-1^_;_ M_w_ = 4,500 g mol^-1^_;_ Đ = 1.18.

P1: [B][Phe]_3_[C_6_]_3_[PEO_2k_-Cy5] **1**

Compound **19** (8.92 mg, 3.28 µmol, 1.0 eq) was dissolved in 110 µL DMF. 120 µL of a 42 mg/mL DIPEA stock solution in DMF (DIPEA: 8.71 µL, 50 µmol, 15 eq) were added and the solution stirred for 15 min at rt. To this, 280 µL of a 12.5 mg/mL NHS-Cy5 stock solution in DMF (NHS-Cy5: 3.5 mg, 4.59 µmol, 1.4 eq) was added and the reaction mixture stirred for 3 d at rt. To remove excess and unconjugated dye, the reaction mixture was diluted with water and dialysed against a 0.015 M NaCl solution for 9 days, followed by dialysis against water for 2 d. The desired dye-conjugate could then be obtained via lyophilization.

The dye-conjugation efficiency was determined via the SEC-UV detector set to the emission maximum of Cy5. With the knowledge of the initially used eq of NHS-Cy5 and the different elution times of NHS-Cy5 and the dye-conjugates, a calculation of the coupling efficiency was possible by comparing the area of the elution peaks. This determination of the coupling efficiency was checked once for this compound via ^1^H-NMR spectroscopy.

**Yield:** 6.47 mg, 2.00 µmol (61%), blue powder.

**Dye content:** 72%

**^1^H-NMR (300 MHz, d_6_-DMSO, 298 K):** δ [ppm] = 8.78 – 8.68 (m, 3H, NH), 8.43 – 8.21 (m, 5H, CH_aromatisch_ & CH=CH_Cy5_), 8.18 – 8.02 (m, 3H, NH), 7.90 – 7.76 (m, 2H, CH_aromatisch,Cy5_), 7.69 – 7.59 (m, 2H m, 2H, CH_aromatisch,Cy5_), 7.38 – 7.10 (m, 17H, CH_aromatisch_ & CH_aromatisch,Cy5_), 6.57 (t, J = 12.4 Hz, 1H, CH=CH_Cy5_), 6.28 (t, J = 13.9 Hz, 2H, CH=CH_Cy5_), 4.71 (q, J = 8.9 Hz, 3H, CH), 4.12 – 3.95 (m, 4H, CH_2_), 3.78 – 3.68 (m, 2H, CH_2_), 3.58 (s, 3H, CH_3,Cy5_), 3.55 – 3.46 (m, 152H, PEO), 3.21 – 2.86 (m, 14H, CH_2_), 1.76 – 1.60 (m, 16H, CH_2,Cy5_ & CH_3.Cy5_), 1.59 – 1.45 (m, 2H, CH_2,Cy5_), 1.43 – 1.28 (m, 8H, CH_2_), 1.28 – 1.08 (m, 16H, CH_2_), 0.83 (t, J = 6.5 Hz, 6H, CH_3_).

**SEC (DMAc + 0.21 wt.% LiCl):** M_n_ = 3,700 g mol^-1^_;_ M_w_ = 4,400 g mol^-1^_;_ Đ = 1.16.

P2: [B][Phe]_3_[C_8_]_3_[PEO_2k_-Cy5] **2**

**2** was synthesized according to the procedure of compound **1**.

**Yield:** 9.71 mg, 2.93 µmol (89%), blue powder.

**Dye content:** 79%

**SEC (DMAc + 0.21 wt.% LiCl):** M_n_ = 4,000 g mol^-1^_;_ M_w_ = 4,400 g mol^-1^_;_ Đ = 1.08.

P3: [B][Phe]_3_[C_10_]_3_[PEO_2k_-Cy5] **3**

**3** was synthesized according to the procedure of compound **1**.

**Yield:** 12.24 mg, 3.60 µmol (96%), blue powder.

**Dye content:** 100%

**SEC (DMAc + 0.21 wt.% LiCl):** M_n_ = 4,600 g mol^-1^_;_ M_w_ = 5,100 g mol^-1^_;_ Đ = 1.09.

P4: [B][Phe]_3_[C_12_]_3_[PEO_2k_-Cy5] **4**

**4** was synthesized according to the procedure of compound **1**.

**Yield:** 10.6 mg, 3.04 µmol (81%), blue powder.

**Dye content:** 87%

**SEC (DMAc + 0.21 wt.% LiCl):** M_n_ = 4,300 g mol^-1^_;_ M_w_ = 4,700 g mol^-1^_;_ Đ = 1.08.

P5: [B][Ala]_3_[C_12_]_3_[PEO_2k_-Cy5] **5**

**5** was synthesized according to the procedure of compound **1**.

**Yield:** 8.88 mg, 2.72 µmol (83%), blue powder.

**Dye content:** 81%

**SEC (DMAc + 0.21 wt.% LiCl):** M_n_ = 4,200 g mol^-1^_;_ M_w_ = 4,500 g mol^-1^_;_ Đ = 1.07.

P6: [B][U]_3_[C_12_]_3_[PEO_2k_-Cy5] **6**

**6** was synthesized according to the procedure of compound **1**.

**Yield:** 6.98 mg, 2.26 µmol (69%), blue powder.

**Dye content:** 100%

**SEC (DMAc + 0.21 wt.% LiCl):** M_n_ = 4,700 g mol^-1^_;_ M_w_ = 4,800 g mol^-1^_;_ Đ = 1.04.

# Characterization

## Cryo transmission electron microscopy (cryoTEM) of assembled SPBs

P1: [B][Phe]_3_[C_6_]_3_[PEO_2k_-Cy5] **1**


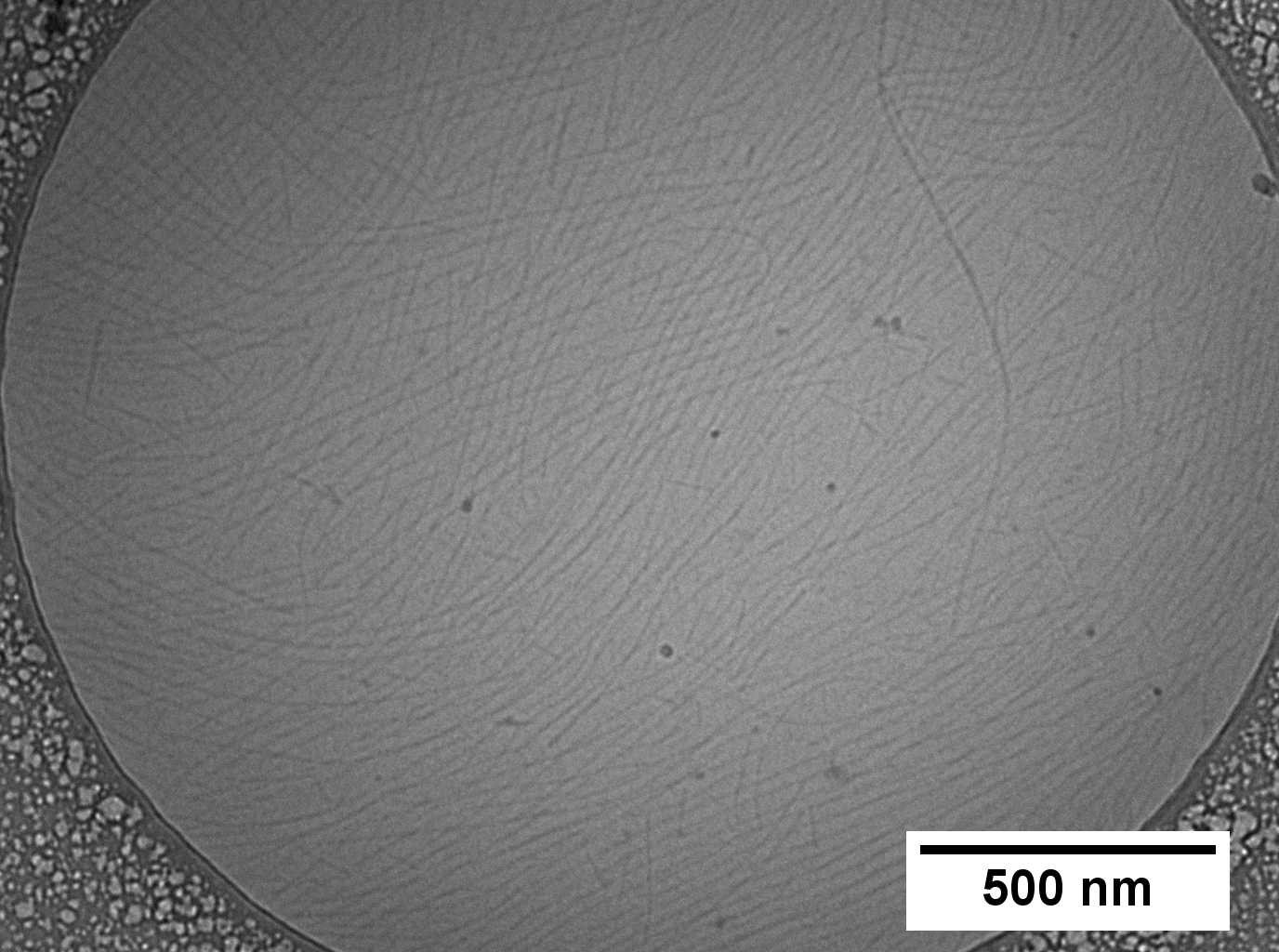


Figure S1. CryoTEM of SPB1 after assembly of P1 from DMF with a final solution concentration of $\boldsymbol{c = 1 mg}\boldsymbol{ml}^{\boldsymbol{-1}}$ in water.

P2: [B][Phe]_3_[C_8_]_3_[PEO_2k_-Cy5] **2**


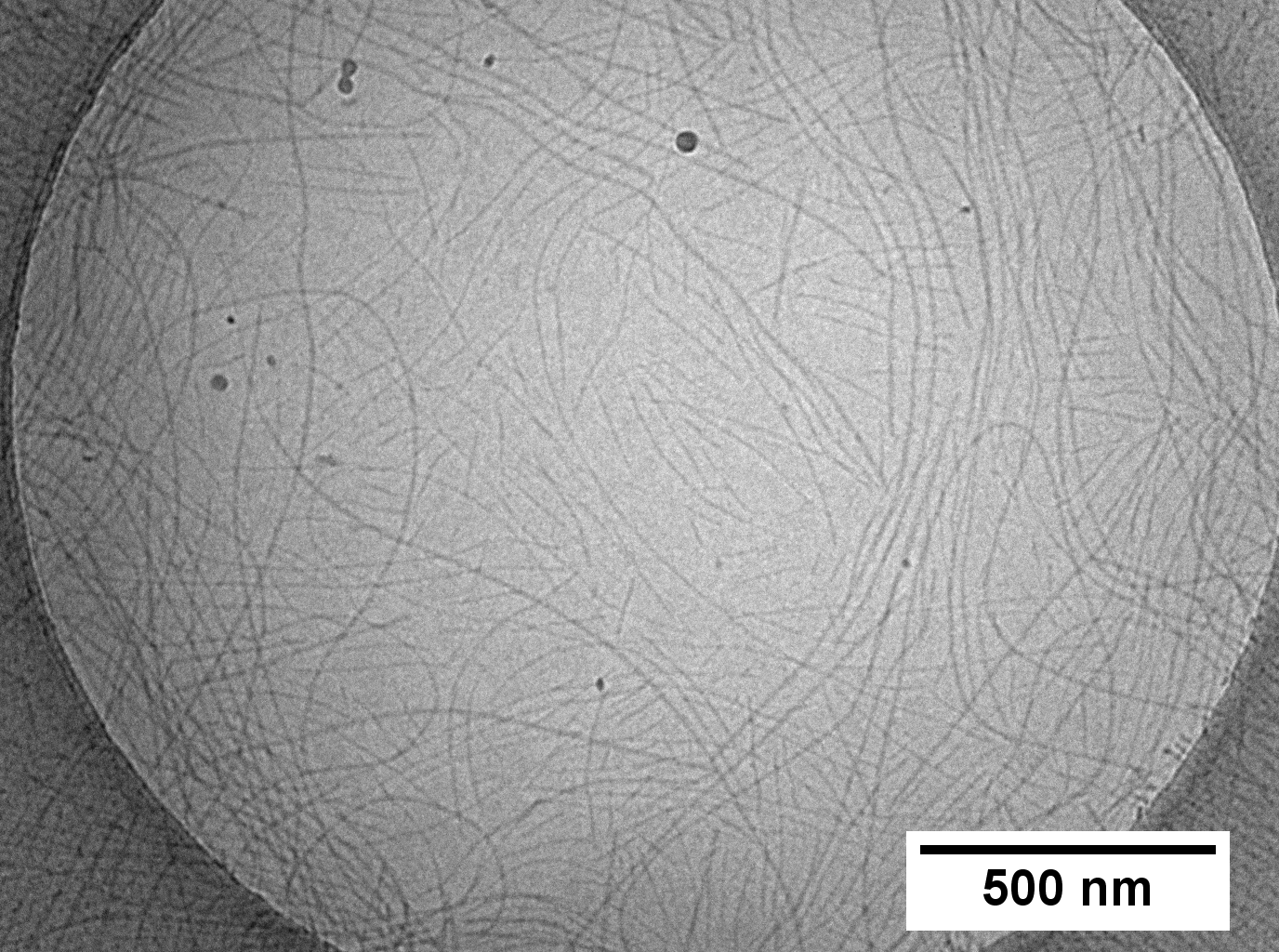


Figure S2. CryoTEM of SPB2 after assembly of P2 from DMF with a final solution concentration of $\boldsymbol{c = 1 mg}\boldsymbol{ml}^{\boldsymbol{-1}}$ in water.

.

P3: [B][Phe]_3_[C_10_]_3_[PEO_2k_-Cy5] **3**


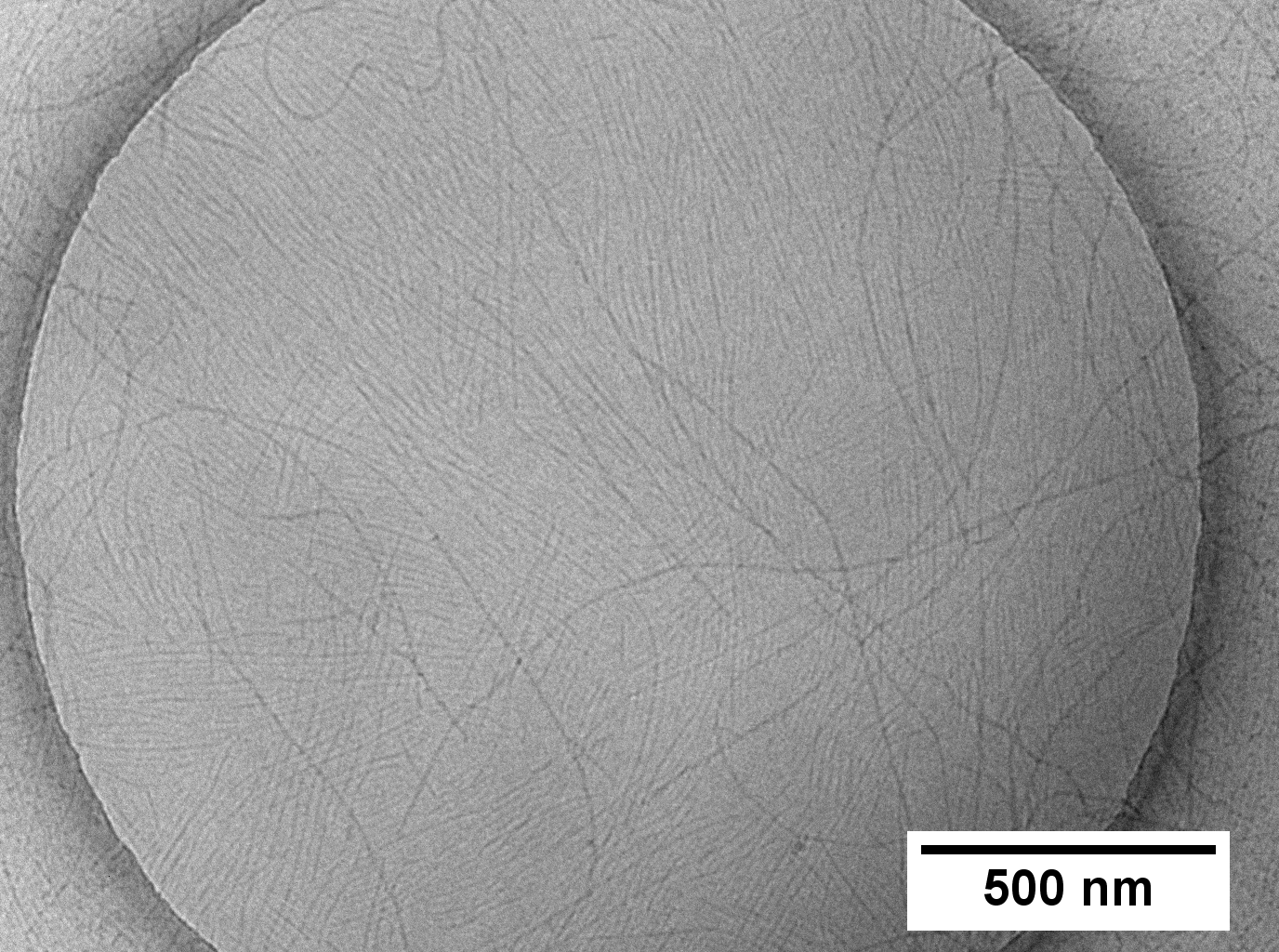


Figure S3. CryoTEM of SPB3 after assembly of P3 from DMF with a final solution concentration of $\boldsymbol{c = 1 mg}\boldsymbol{ml}^{\boldsymbol{-1}}$ in water.

P4: [B][Phe]_3_[C_12_]_3_[PEO_2k_-Cy5] **4**


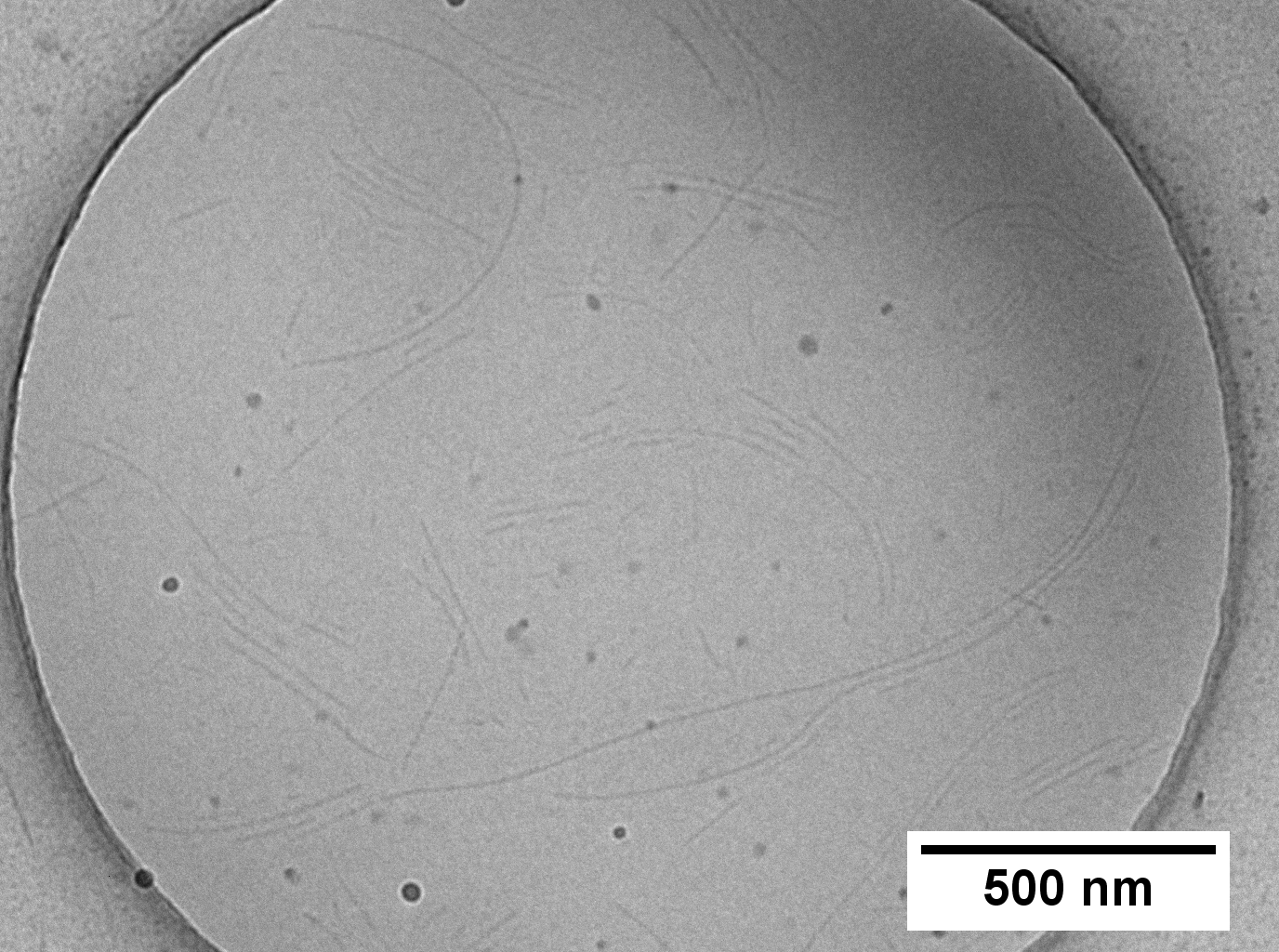


Figure S4. CryoTEM of SPB4 after assembly of P4 from DMF with a final solution concentration of $\boldsymbol{c = 1 mg}\boldsymbol{ml}^{\boldsymbol{-1}}$ in water.

P5: [B][Ala]_3_[C_12_]_3_[PEO_2k_-Cy5] **5**


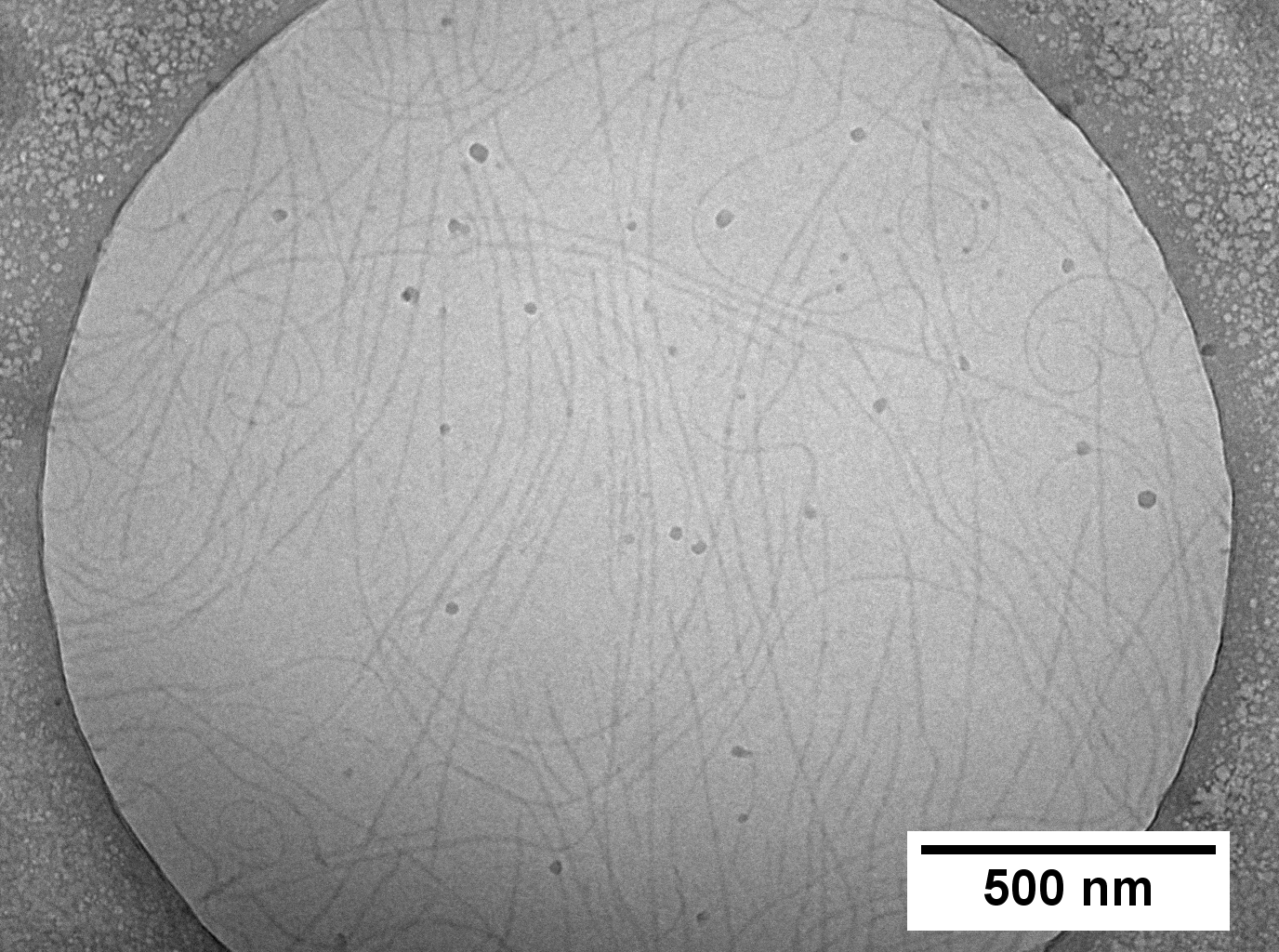


Figure S5. CryoTEM of SPB5 after assembly of P5 from DMF with a final solution concentration of $\boldsymbol{c = 1 mg}\boldsymbol{ml}^{\boldsymbol{-1}}$ in water.

P6: [B][U]_3_[C_12_]_3_[PEO_2k_-Cy5] **6**


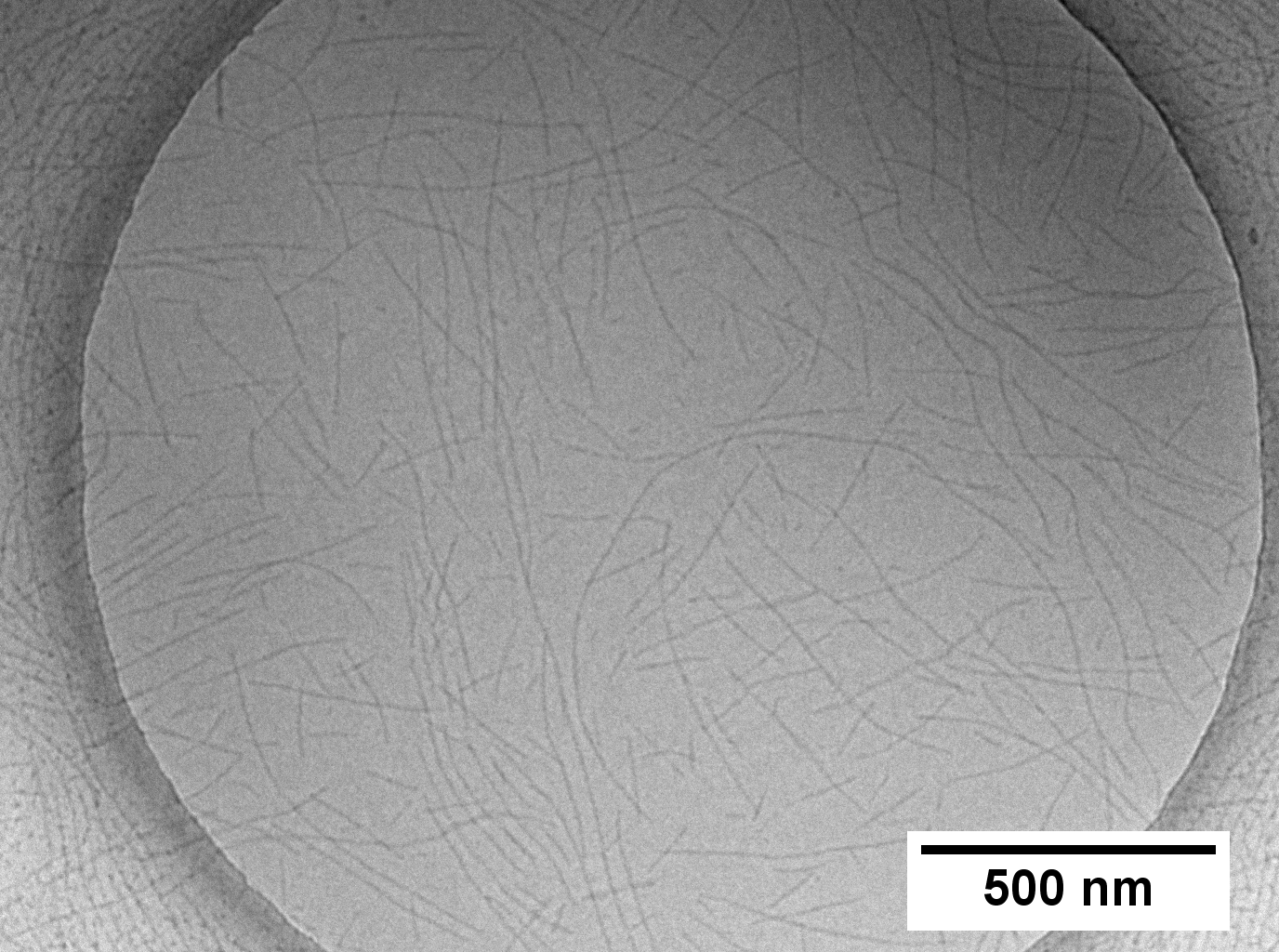


Figure S6. CryoTEM of SPB6 after assembly of P6 from DMF with a final solution concentration of $\boldsymbol{c = 1 mg}\boldsymbol{ml}^{\boldsymbol{-1}}$ in water.

## Analytical ultracentrifugation (AUC)


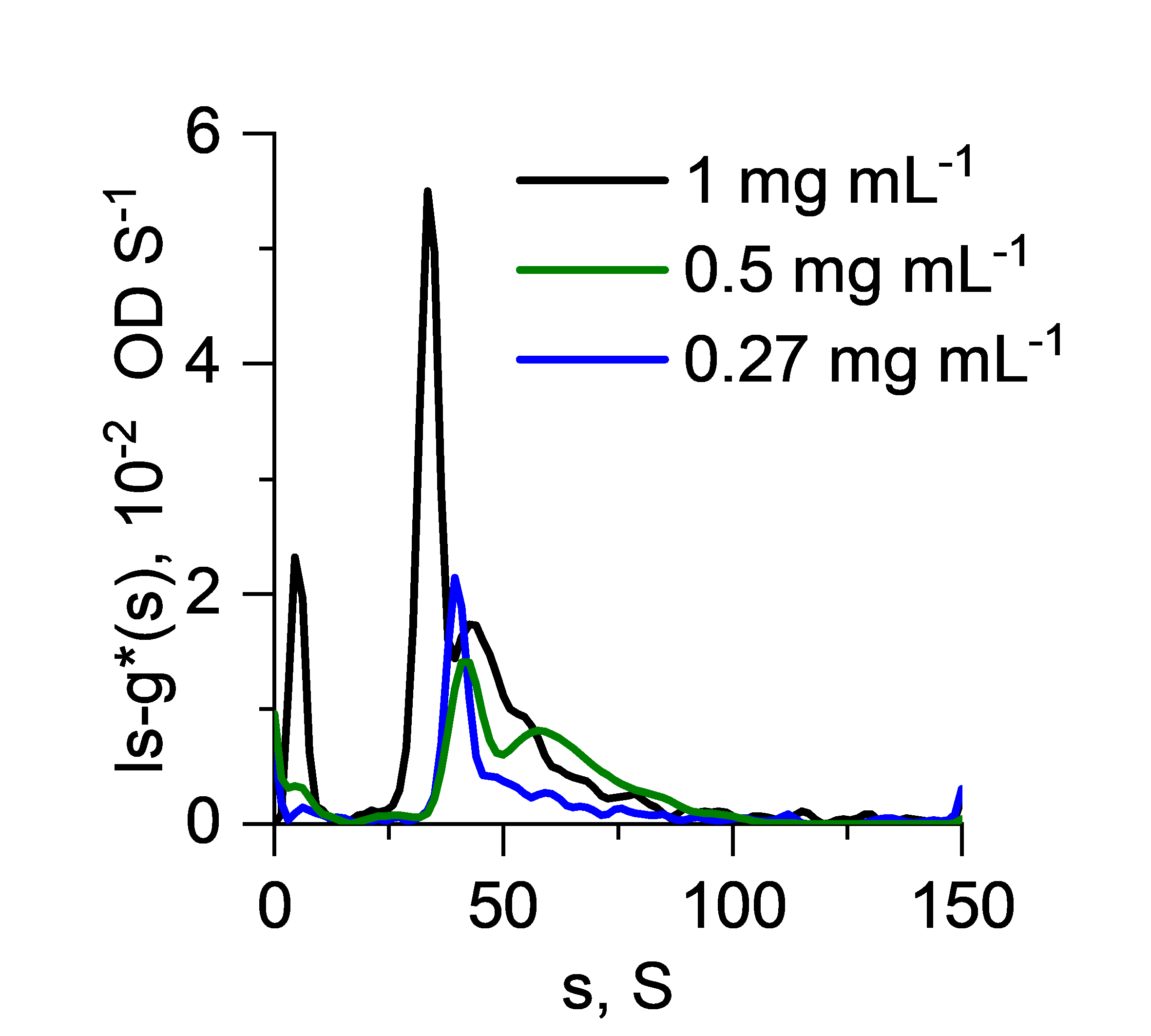


**Figure S7**. Differential distributions of sedimentation coefficients of SPB4 at varying concentrations and a temperature of $T = 20 ^{\circ}C$.


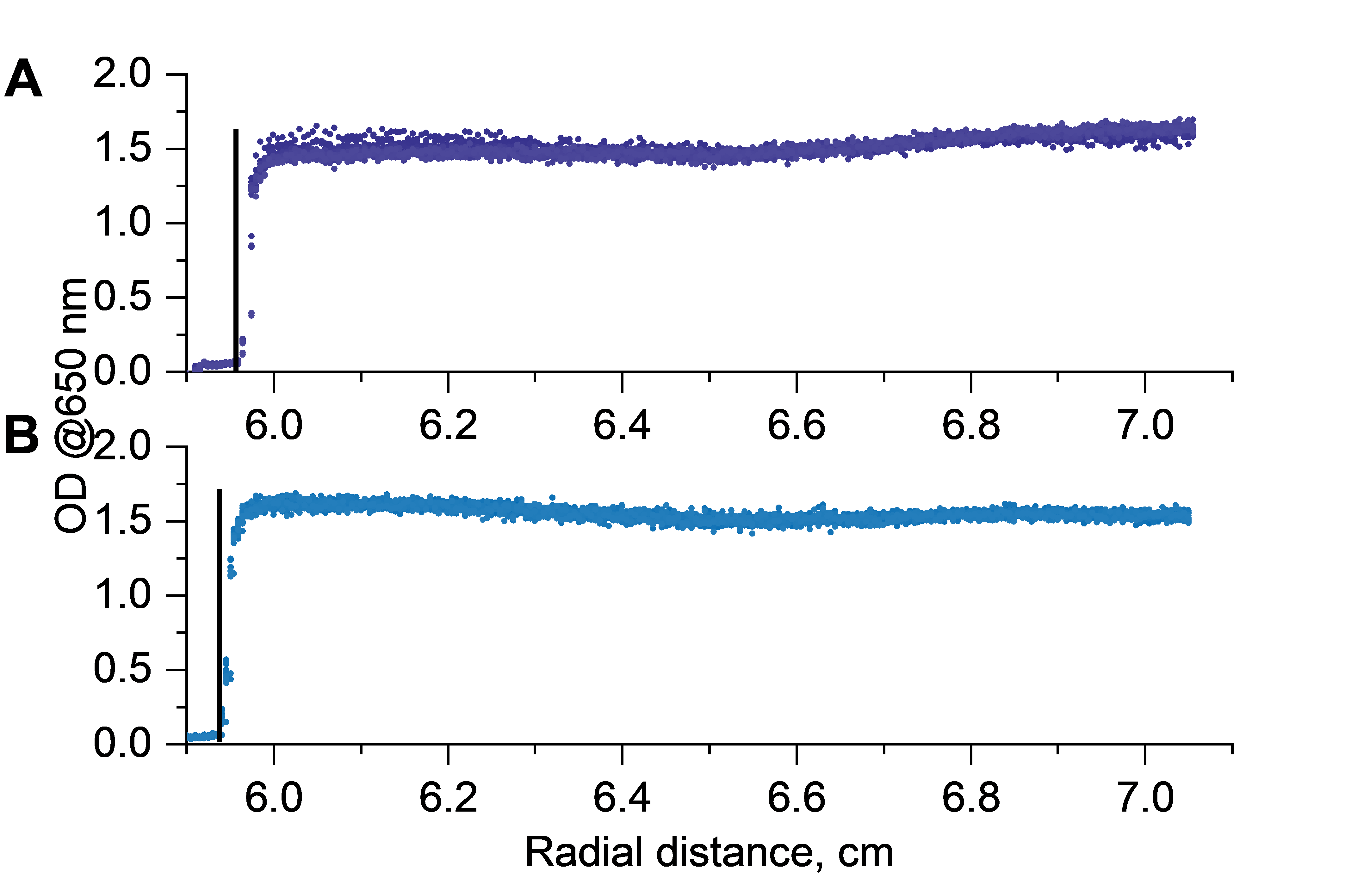


Figure S8. Sedimentation velocity profiles of Cy5 (used for labelling) in water at (A) 20,000 rpm and (B) 42,000 rpm obtained from the absorbance detection module at the wavelength of $\boldsymbol{\lambda}\mathbf{= 650}\boldsymbol{nm}$. Experiments were performed at a temperature of $\boldsymbol{T}\boldsymbol{= 20 ^{\circ}}\boldsymbol{C}$. The black solid vertical lines indicate positions of the menisci.


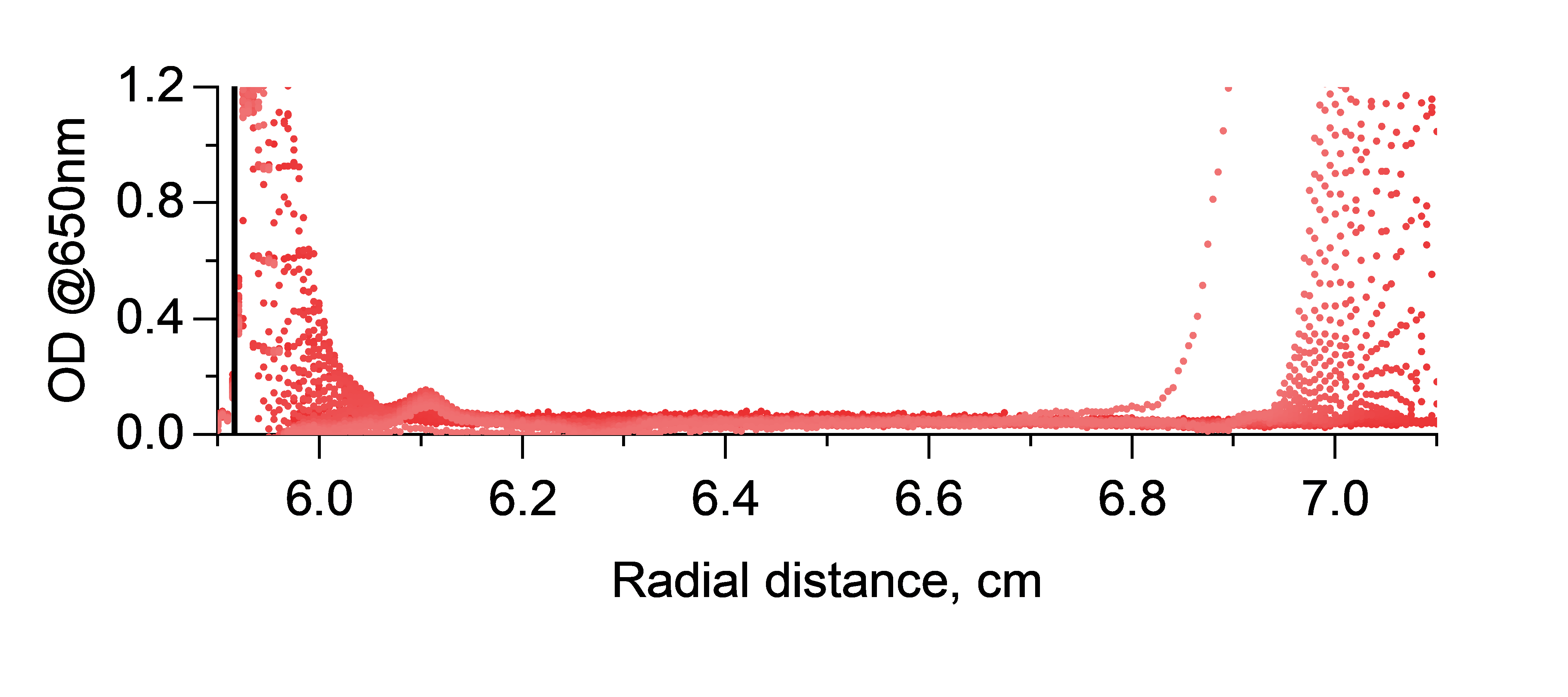


**Figure S9**. Sedimentation velocity experiments of HS at 20,000 rpm. The sedimentation velocity profiles were monitored at a wavelength of $\lambda= 650 nm$. The experiment was performed at a temperature of $T = 20 ^{\circ}C$. The black solid vertical line indicates the position of the meniscus.


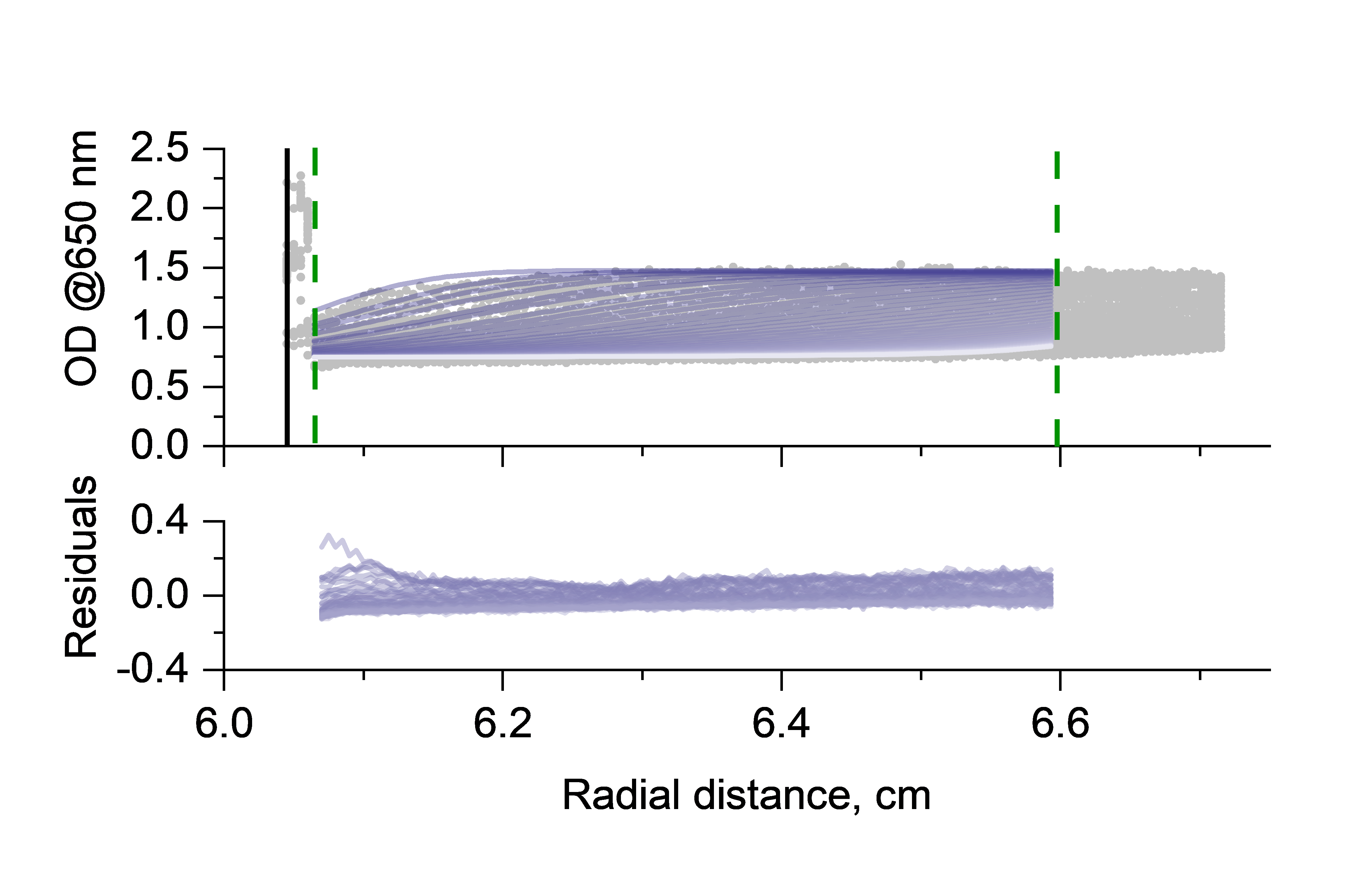


**Figure S10**. Sedimentation velocity profiles of Cy5 (used for labelling) ($c = 2 g {ml}^{-1}$) in HS at 20,000 rpm. Gray dots are the measured data and colored lines are the result from the $c(s)$analysis with the residuals shown in the bottom. The sedimentation velocity profiles were obtained with the absorbance detection module at a wavelength of $\lambda= 650 nm$. The experiment was performed at a temperature of $T = 20 ^{\circ}C$. The black solid vertical line indicates the position of the meniscus, and the green dashed vertical lines indicate the region where modelling of the data was performed.


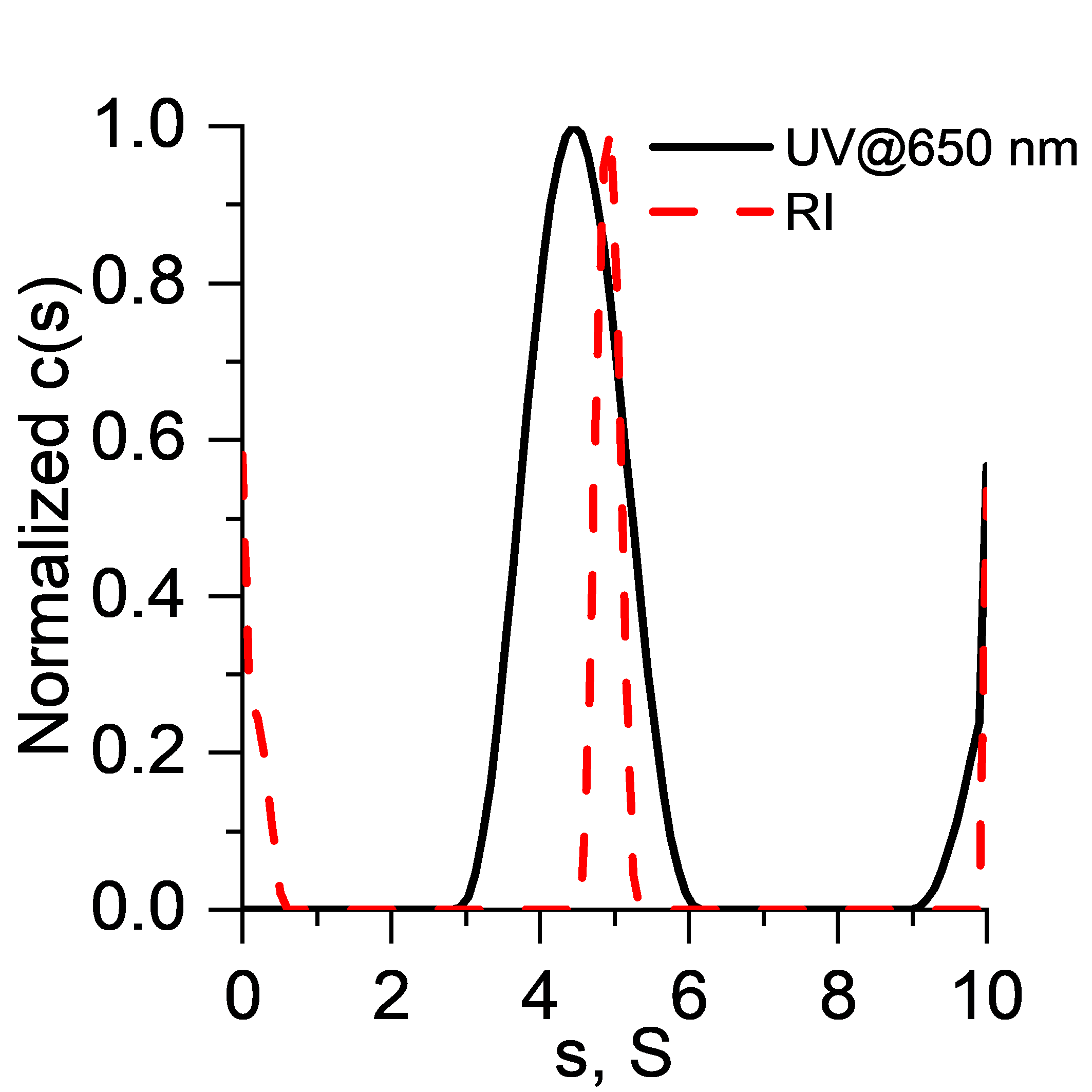


**Figure S11**. Normalized differential distributions of sedimentation coefficients, $c(s)$, obtained by modelling sedimentation velocity data of Cy5 (used for labelling) in solution with HSA (Figure S9) recorded via RI and UV absorption optics (the latter at a wavelength of $\lambda= 650 nm$) at a rotor speed of 20,000 rpm. The experimental data for modelling were acquired at a temperature of $T = 20 ^{\circ}C$.


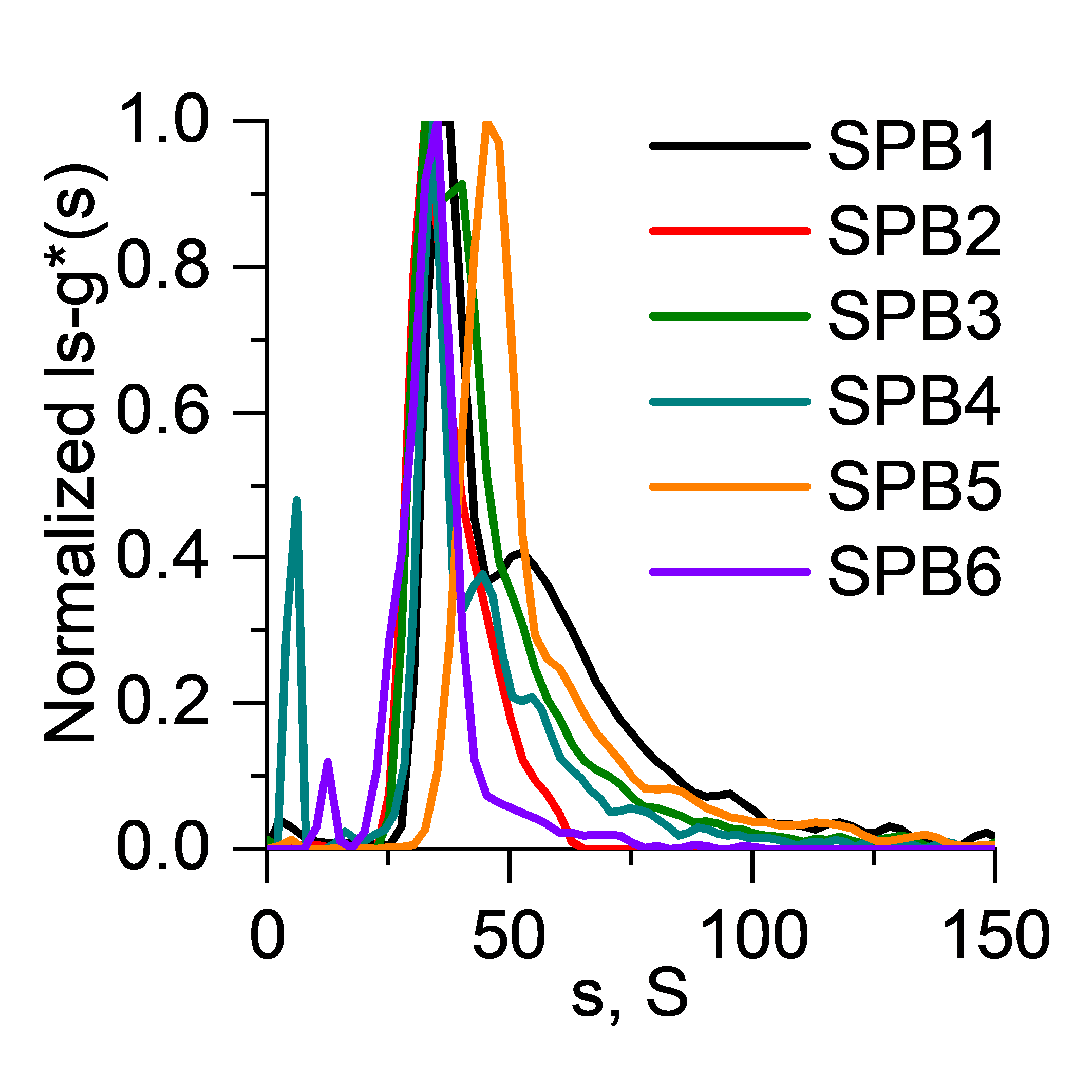


**Figure S12**. Normalized differential distributions of sedimentation coefficients, $ls-g^{*}(s)$, of all studied samples in water from sedimentation velocity experiments at 20,000 rpm with UV absorption optics.


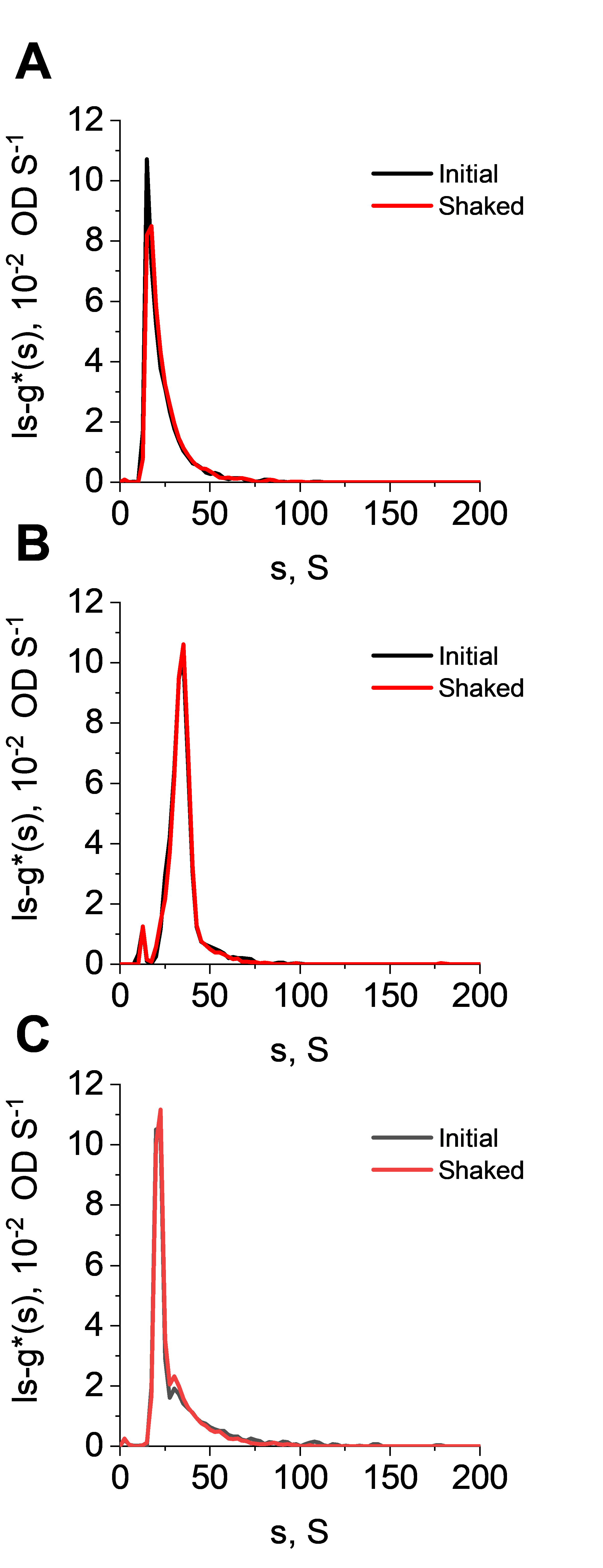


**Figure S13**. Differential distributions of sedimentation coefficients, $ls-g^{*}(s)$, of sample (A) SPB3, (B) SPB5, (C) SPB6 in water. The experimental data was obtained with the absorbance detection optics at the wavelength of $\lambda= 650 nm$ and a rotor speed of 20,000 rpm. The experimental data were acquired at a temperature of $T = 37 ^{\circ}C$.


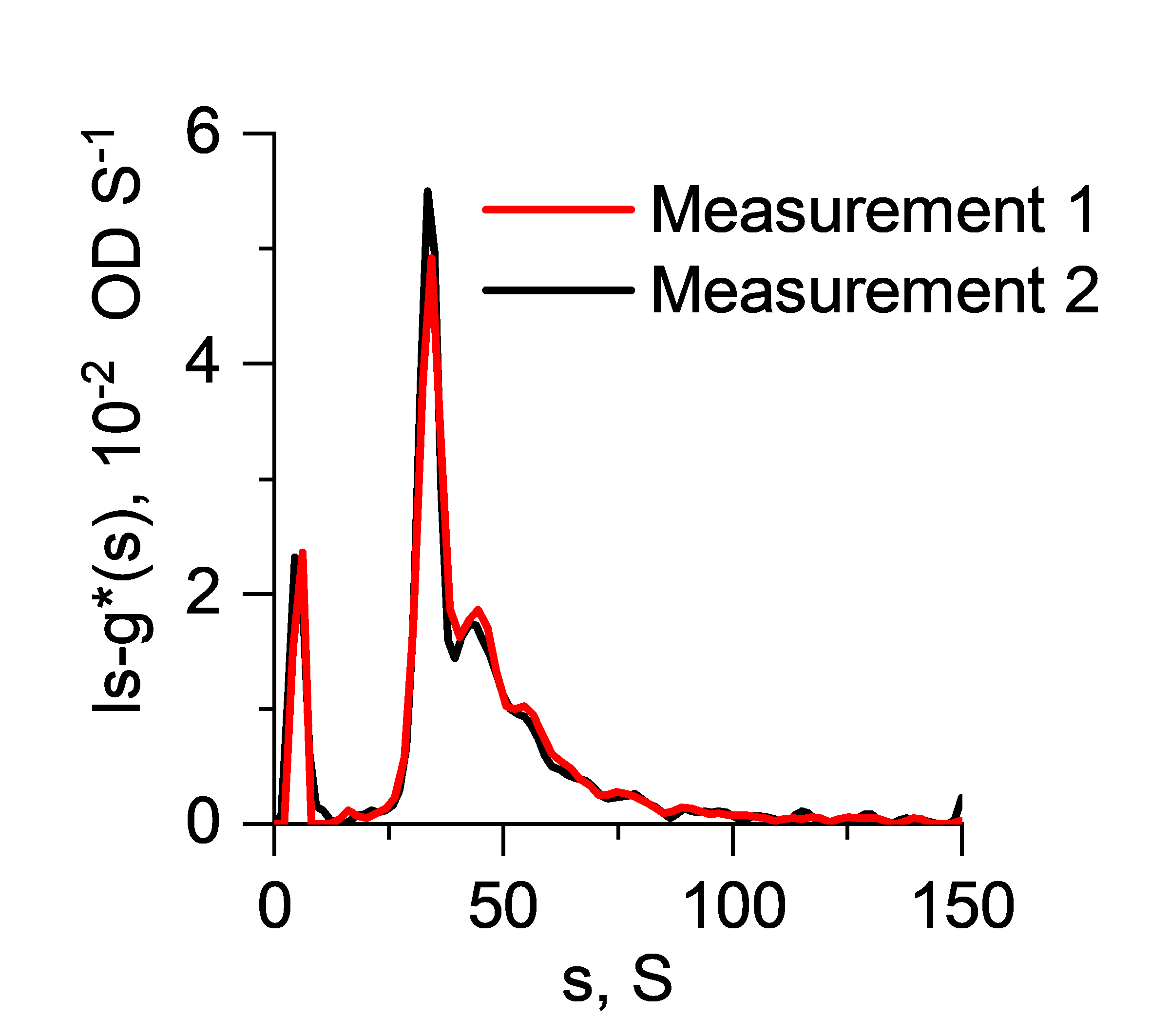


**Figure S14.** Differential distributions of sedimentation coefficients of SPB4 obtained in separate experiments in water with a 4-month time gap at a temperature of $T = 20 ^{\circ}C$.

**
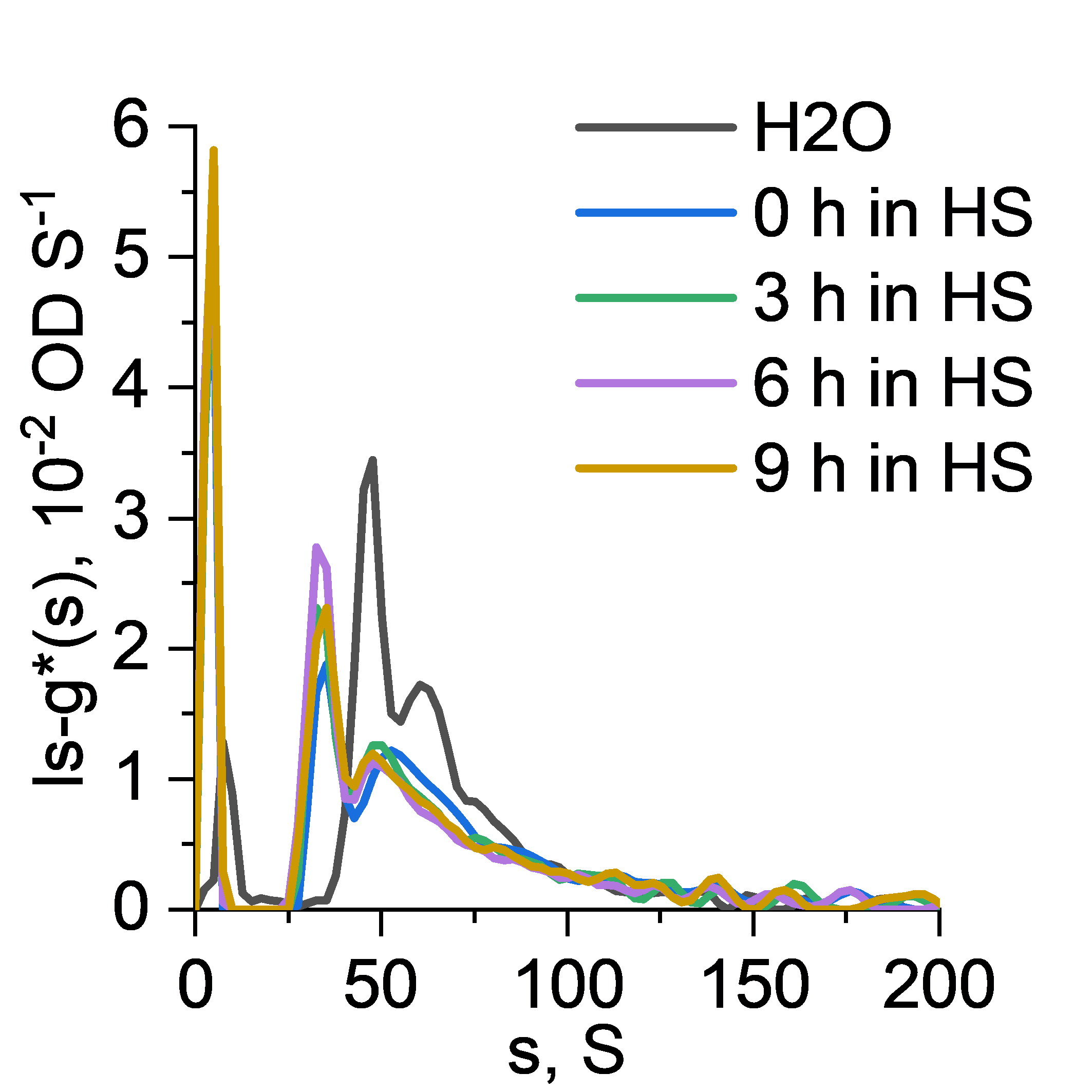
**

**Figure S15**. Differential distributions of sedimentation coefficients, $ls-g^{*}(s)$, of sample SPB4 in water and at different storage time in HS. The experimental data was obtained with the absorbance detection optics at a wavelength of $\lambda= 650 nm$ and a rotor speed of 20,000 rpm. Experiments were performed at a temperature of $T = 37 ^{\circ}C$.


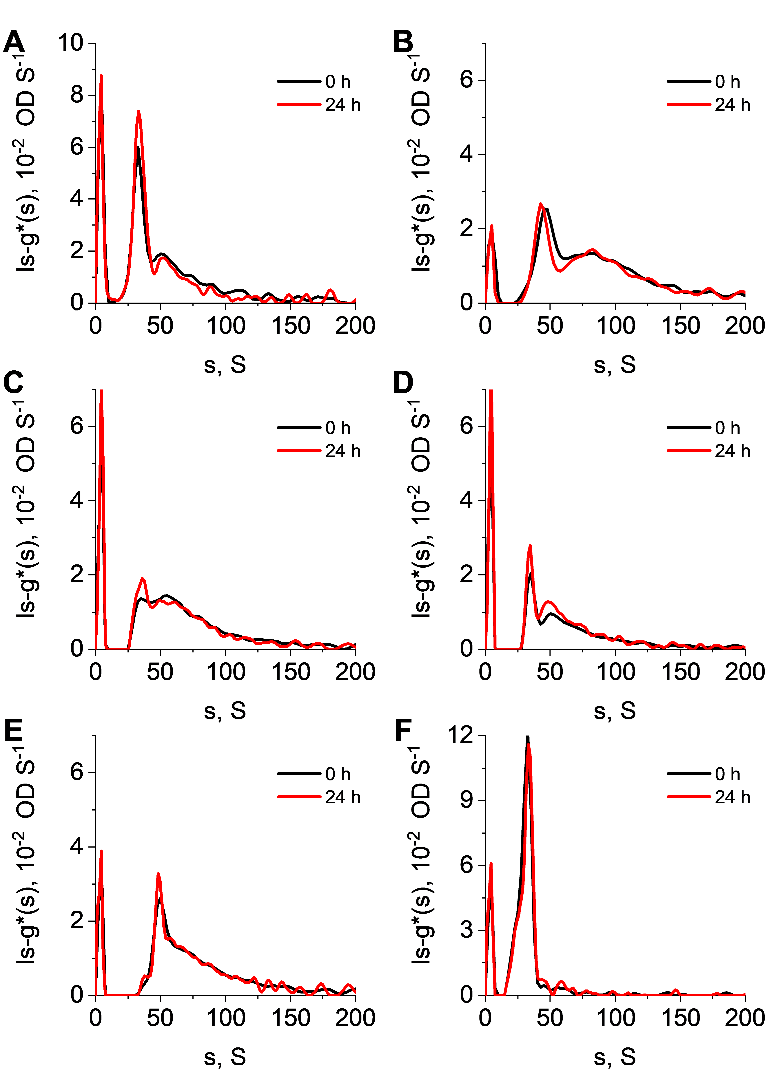


**Figure S16**. Differential distributions of sedimentation coefficients, $ls-g^{*}(s)$ of (A) SPB1, (B) SPB2, (C) SPB3, (D) SPB4, (E) SPB5, and (F) SPB6 obtained after different storage times in HS. The experimental data was obtained with the absorbance detection optics at the wavelength of $\lambda= 650 nm$ and a rotor speed of 20,000 rpm. Experimental data were acquired at a temperature of $T = 37 ^{\circ}C$.

**
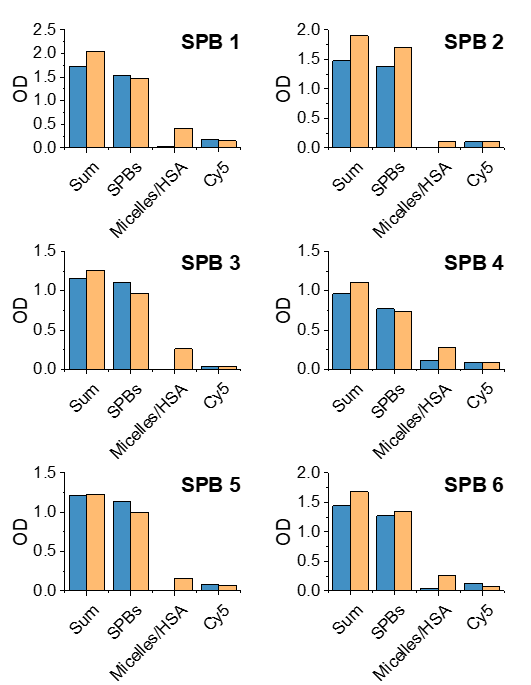
**

**Figure S17**. Bar charts from the OD level at $\lambda= 650 nm$ of the different components all samples in water (blue bars) and after 24 hours of storage at $T = 37$ °C in HS before the respective AUC experiments (orange bars).

**Table S1.** Measured solvent properties at different temperatures.

| $Solvent$ | $\eta_{0}$*,* $mPa s$ | $\rho_{0}$*,* $g {cm}^{-3}$ |
| --- | --- | --- |
| H_2_O, $T = 20 ^{\circ}C$ | 1.002 | 0.99827 |
| H_2_O, $T = 37 ^{\circ}C$ | 0.735 | 0.99337 |
| H_2_O/D_2_O, $T = 20 ^{\circ}C$ | 1.225 | 1.06914 |
| Human serum, $T = 37 ^{\circ}C$ | 0.983 | 1.00899 |

**Table S2**. Determined partial specific volumes, $\upsilon$, of the studied samples.

| $Sample$ | $\upsilon, {cm}^{3}g^{-1}$ |
| --- | --- |
| SPB1 | 0.84 |
| SPB2 | 0.87 |
| SPB3 | 0.84 |
| SPB4 | 0.87 |
| SPB5 | 0.86 |
| SPB6 | 0.85 |

References

[1] T. Klein, H. F. Ulrich, F. V. Gruschwitz, M. T. Kuchenbrod, R. Takahashi, S. Fujii, S. Hoeppener, I. Nischang, K. Sakurai, J. C. Brendel, *Polymer Chemistry* **2020**, 11, 6763.

[2] F. V. Gruschwitz, *PhD Thesis*, Friedrich Schiller University, Jena, **2021**.
